# From Claims to Violence: Signaling, Outbidding, and Escalation in Ethnic Conflict

**DOI:** 10.1177/0022002721996436

**Published:** 2021-03-02

**Authors:** Manuel Vogt, Kristian Skrede Gleditsch, Lars-Erik Cederman

**Affiliations:** 1 4919 University College London, United Kingdom; 2 2591 University of Essex, Colchester, United Kingdom; 3 Peace Research Institute Oslo, Norway; 4 ETH Zürich, Zürich, Switzerland

**Keywords:** civil wars, internal armed conflict, conflict, rebellion

## Abstract

Do radical political demands increase the risk of ethnic civil conflict? And why do ethnic movements make radical demands in the first place? We contend that when movements are fragmented, individual organizations use far-reaching claims relative to the status quo to attract attention from the government, boost intra-organizational discipline, and outbid rivals. Yet, such radical claims also increase the risk of conflict escalation. We test our arguments at both the ethnic group and organizational levels, using a new dataset on ethno-political organizations and their political demands. Our results show that the scope of demands increases the more organizations exist within an ethnic movement and that radical demands increase the risk of civil conflict onset. This effect is specific to the dyadic government-movement interaction, irrespective of other ethnic groups in the country. Moreover, at the organizational level, radicalization in demands increases the likelihood that an organization becomes engaged in civil conflict.

## Introduction

In October 2017, the latent conflict between the Cameroonian government and Anglophone opposition groups escalated into deadly violence, causing hundreds of fatalities. After initial calls for language and administrative rights in the Anglophone regions were resisted by the government, the dissidents made further demands for political autonomy and an independent Anglophone state of “Ambazonia,” provoking heavy-handed government repression. Do radical political demands increase the risk of ethnic civil conflict? And why do ethnic movements make radical demands in the first place? Recent research highlights the impact of actor fragmentation (Cunningham 2013) and organizational rivalries (Cunningham, Bakke, and Seymour 2012; Krause 2014; Pearlman 2008/09) on conflict dynamics, but has not analyzed in depth how radicalization in opposition demands intervenes in these processes. Similarly, existing studies show an effect of inter-group inequality on ethnic civil conflict (Cederman, Gleditsch, and Buhaug 2013; Stewart 2008), but do not consider how claims made by marginalized groups affect the escalation process leading from inequality to conflict outbreak.

To address this gap, we develop a theory of conflict escalation in ethno-political mobilization that highlights the importance of claims as signaling devices within opposition movements and in their interaction with state governments. With a higher number of organizations within an ethnic movement, individual organizations have incentives to make radical claims to attract attention and possible concessions from the government. In the face of movement-internal competition, radical claims also serve to boost intra-organizational discipline and outbid contenders for the same popular support. Yet, such radical claims increase the risk of violent conflict escalation through the perceived threat to the government, by empowering hardliners on the government side, and by provoking repressive measures that may fuel anti-government violence.

Our study is the first to measure the scope of political demands at the level of individual organizations, including both violent and non-violent organizations, in a globally representative sample. We define the scope of demands as a function of distance from the status quo on two different dimensions: governmental power and territorial rights. The larger this distance, the more radical the demand is. We introduce a new dataset on ethno-political organizations, EPR-Organizations (EPR-O), which covers a random sample of forty countries over the period 1946 to 2013 and identifies individual organizations representing groups in the Ethnic Power Relations (EPR) dataset (Cederman, Wimmer, and Min 2010; Vogt et al. 2015), as well as their distinct claims. These data allow us to examine the causes and consequences of radical demands at the levels of both ethnic groups and organizations. Since EPR-O covers both violent and non-violent actors, we can track the political demands of organizations that never become involved in violence, as well as organizations’ claims *before* they engage in violence, and thus evaluate how the scope of demands affects the risk of ethnic civil conflict.

Our empirical analysis of the roots of radicalization shows that the scope of demands increases as a function of the number of organizations within an ethnic movement. This implies that although most comparative research conceives of radicalization as observed escalation in violence (e.g., Bloom 2004; Cunningham, Bakke, and Seymour 2012; Lawrence 2010), claims constitute an important element of inter-organizational outbidding dynamics, as highlighted in many case studies (e.g., Haines 1995; Kaufman 1996). Moreover, the risk of ethnic civil conflict is higher the more radical the demands of an ethnic movement relative to the status quo. We also find that the risk of civil conflict increases due to escalation in the dyadic government-movement interaction rather than potential future imitators. Finally, we find evidence of an organization-specific effect of radicalization; as demands become more radical, an organization is more likely to become engaged in civil conflict. Together, these findings provide new insights into the causal pathways that lead from previously identified risk factors to ethnic civil conflict.

## Ethnic Mobilization, Actor Fragmentation, and Civil Conflict

Although many scholars emphasize how ethnic mobilization can fuel violent conflict (Brancati 2006; Horowitz 1985; Rabushka and Shepsle 1972), most ethno-political disputes do not give way to violence. Existing research has considered both structural and actor-centered approaches to explain non-violent and violent conflict and how the former may escalate into the latter (Chenoweth and Ulfelder 2017; Vogt 2019). Yet, the role of specific claims made by political actors in such escalation has largely been ignored.

For instance, one important strand of research argues that politically excluded or economically marginalized groups are especially likely to rebel against the state (Cederman, Gleditsch, and Buhaug 2013; Stewart 2008). These arguments postulate a process of group mobilization running from inequality to violence (e.g., Cederman, Gleditsch, and Buhaug 2013, 44-48), but while acknowledging the importance of organizations in engendering collective action (e.g., Vogt 2019, 61-73), they do not consider the impact of organizational claims and within-group competition on the risk of escalation. From this perspective, claims advanced on behalf of ethnic groups should simply reflect the grievances, which follow from structural inequalities. Other studies treat opposition claims as a function of mobilization capacity and bargaining leverage (e.g., Cetinyan 2002; Jenne, Saideman, and Lowe 2007). For example, Cetinyan (2002) argues that under perfect information, an ethnic movement should make demands on the state proportional to its capability. However, there are often important information asymmetries between government and opposition movements (Walter 2009), and opposition claims can play an important role in creating or exploiting such asymmetries.

Recent work extends bargaining models beyond unitary actors and considers the role of internal divisions within the main antagonists. One key finding is that fragmented political movements are more likely to engage in violence against the state than unitary movements because fragmentation raises uncertainty and exacerbates commitment problems (Cunningham 2013). While providing crucial insights into how violence is affected by power struggles and alliances between and within organizations (Christia 2012; Krause 2014; Pearlman 2008/09), these studies also disregard the specific claims made by conflict actors. This makes it difficult to directly test many proposed accounts of conflict escalation. To illustrate, Cunningham (2013, 661) notes that “[b]argaining breaks down when the state is unable or unwilling to satisfy the demands of the opposition.” This raises the question of *what* demands the state cannot or will not concede.

Moreover, claims can also play a crucial role in movement-*internal* signaling. For instance, existing studies conceptualize outbidding as organizations’ escalating use of violence (e.g., Bloom 2004; Cunningham, Bakke, and Seymour 2012; Lawrence 2010). Yet, theories of ethnic outbidding suggest that organizations can also outbid each other through the scope of their claims (e.g., Horowitz 1985, 185-228; Kaufman 1996). Similarly, social movement scholars have highlighted the role of movement factions’ objectives and rhetoric in their analyses of “radical flanks” (e.g., Gamson 1975, 46-49; Haines 1995). Some studies have collected data on the content of conflicts, such as the Religion and Armed Conflict data (Svensson and Nilsson 2018), or the motivations and ideologies of non-state actors, such as the MAROB dataset (Wilkenfeld, Asal, and Pate 2011). Yet, these works typically assume certain claims a priori to be particularly prone to violence, especially religious or territorial demands (Asal and Rethemeyer 2008; Hassner 2009; Svensson 2007; Toft 2002, 2007; Walter 2006). This overlooks important variation in the scope of demands within the same type of conflict issue.

In contrast, we argue that the most relevant factor is how far political demands depart from the status quo, independent of the particular issue at stake. We define demands as radical when they call for far-reaching changes to the status quo. Thus, we propose a relational operationalization of the scope of demands.^1^ For example, a demand for more regional autonomy is more radical in a highly centralized state than in a federal state, and demands for inclusion that are radical in a highly exclusionary state may represent the status quo in another state. We apply our operationalization to a theory of how radical claims affect bargaining with the state in ethno-political conflicts.

## Signaling and Outbidding: A Claim-based Theory of Ethnic Conflict Escalation

We develop a two-stage argument about the crucial signaling function of claims in ethnic conflict that elucidates, first, why organizations expect radical demands to deliver club benefits in the face of movement-internal competition and, second, how these same signals—though potentially beneficial for individual organizations—can produce negative externalities for the whole movement in the form of armed civil conflict. We distinguish between the overall movement representing an ethnic group and individual organizations, which are typically concerned with both public goods for their ethnic constituency and club goods that exclusively accrue to themselves (Cunningham, Bakke, and Seymour 2012; Krause 2014). In this setting, organizations use claims to signal strength and resolve to three key audiences: the state government, their members, and their broader ethnic constituency.

Since opposition organizations’ interactions with the state government are fraught by information asymmetries, they typically have incentives to exaggerate their strength to obtain more concessions from the government (Walter 2009, 249-50). More radical demands can help an organization appear more committed in bargaining with the state. Moreover, political demands are a core ingredient of ideology and, therefore, play a key role in both mobilizing popular support and establishing organizational cohesion. Ideological doctrines help mobilize individuals for collective action by distinguishing in-groups from out-groups, establishing goals, and providing a road map for action (Costalli and Ruggeri 2015; Drake 1998). Within organizations, they also serve to homogenize individual motivations, develop internal control mechanisms, and, thus, promote intra-organizational discipline (Gutiérrez Sanín and Wood 2014; Thaler 2012). Since radical demands are likely to sharpen ideological profiles, ethno-political organizations may advance such demands in order to boost external support and internal cohesion.^2^

These incentives should increase with a higher number of different organizations representing the same ethnic group.^3^ First, vis-à-vis the government, radical demands can allow individual organizations to gain prominence in bargaining, compared to other organizations, and secure individual benefits. Second, in the competition for popular support within the movement’s constituency, radical demands allow organizations to distinguish themselves from movement-internal competitors and portray themselves as the most committed group representatives (Horowitz 1985, 185-228; Kaufman 1996; Rabushka and Shepsle 1972). Similar to the centrifugal force affecting political candidates in party-internal contests (e.g., Adams and Merrill 2008; Owen and Grofman 2006), increasing competition over the same “market” of supporters incentivizes ethno-political organizations to send more radical signals. For example, Euskadi Ta Askatasuna (ETA, Homeland and Liberty) and its political wing Herri Batasuna (Popular Unity) have rejected any form of autonomy short of full independence, thereby distancing themselves from the mainstream Basque Nationalist Party, which accepts an autonomous Basque province within Spain.

Finally, the emergence of competitors representing the same ethnic constituency also threatens organizations’ internal cohesion by raising the specter of members’ defection to rival organizations. For example, competition over recruits has been a key element of rivalry between different Islamist organizations in Somalia (Ahmad 2016). For the purposes of internal communication, then, making radical demands that accentuate their ideological profile can help organizations “distinguish themselves from rival organizations, allowing for long-term internal cohesion in the face of the enemy” (Ugarriza and Craig 2013, 468). Therefore, we expect the scope of ethnic group demands to increase as a function of the number of organizations representing the group.^4^ This first stage of our argument is expressed in the following hypothesis:

**H1:** The higher the number of organizations representing an ethnic group, the greater the scope of the ethnic movement’s demands.

However, while benefiting individual organizations, radical demands also increase the risk of bargaining failure with the state and escalation to violence. There are at least two mechanisms that lead from radical demands to civil conflict outbreak. First, opposition demands affect the government’s threat perception and, by extension, resort to repression (Davenport 2007). Opposition movements with demands far away from the status quo pose a greater threat to the current order, and governments have reason to assume that movements will escalate toward even more radical demands in the future. This undermines the confidence of the government in the willingness of the movement’s representatives to compromise and furthers the cause of hardliners on the government side who oppose negotiations. Moreover, governments are likely to perceive movements with radical demands as prone to radical action (Abrahms 2012). This may incite heavy-handed repression, which often escalates anti-government mobilization (Lichbach 1987; Moore 1998). This direct effect of the scope of demands on civil war risk is captured in the following hypothesis:

**H2a:** The risk of armed civil conflict increases with the scope of an ethnic movement’s demands.

The second mechanism leading from radical demands to civil conflict outbreak runs through the government’s fear of setting precedents. Governments are particularly wary of giving in to far-reaching demands if they perceive a risk that others will make similar demands in the future (Toft 2002; Walter 2006). The threat of future demands increases the government’s resolve to block far-reaching demands at the present. This effect is conditional as it depends on the existence, and the number, of potential future imitators. Governments are likely to be more intransigent if they have more potential future challengers (Walter 2006), which in turn increases the risk of violent conflict escalation. Thus, one can extend the dyadic government-movement interaction to the role of other ethnic movements in the country.

**H2b:** The likelihood that radical demands of an ethnic movement lead to armed civil conflict increases with the number of other ethnic groups in the state.

Finally, at the organizational level, claim-based radicalization should have a direct impact on the risk of civil war as radicalizing organizations may turn to political violence. As Jenne (2007, 15) argues, ethnic leaders’ claims lock organizations into mobilization, and increasingly radical demands raise their reputation costs from backing down later. Radicalizing organizations might be pushed toward violent action because they fear to jeopardize their standing within the movement and its constituency if they do not follow through with their claims. Hence, once introduced, radical demands often develop a life of their own, torpedoing the bargaining process and increasing the risk of violence between governments and challengers. This organization-level effect of radicalization leads us to our last hypothesis:

**H3:** Radicalization of claims increases the likelihood that an organization becomes engaged in armed civil conflict.

## The EPR-Organizations (EPR-O) Dataset

We test our theoretical arguments with a new dataset on ethno-political organizations called EPR-Organizations. EPR-O identifies individual political organizations representing ethnic groups listed in the *Ethnic Power Relations* (EPR) dataset (Cederman, Wimmer, and Min 2010; Vogt et al. 2015). It currently includes a stratified random sample of twenty conflict and twenty non-conflict countries, listed in Table 1, from 1946 (or independence) to 2013.^5^ The dataset focuses on organizations that represent groups at the national level, such as political parties, NGOs, and self-determination organizations. The concept of an organization is defined relatively broadly as any named non-state entity that recruits members and makes political claims. Overall, the dataset contains 668 individual organizations that represent 158 different ethnic groups (see Online Appendix I).

**Table 1.** EPR-Organizations Sample.

Ethnic Civil Conflict Countries	Countries without Ethnic Civil Conflicts
Angola	Algeria
Azerbaijan	Australia
Bangladesh	Belgium
Bosnia and Herzegovina	Botswana
Burundi	Brazil
China	El Salvador
Iraq	Guinea-Bissau
Israel	Lithuania
Macedonia	Madagascar
Myanmar	Malawi
Pakistan	Malaysia
Russia	Mongolia
South Sudan	Mozambique
Spain	Namibia
Sri Lanka	Paraguay
Tajikistan	Peru
Trinidad and Tobago	Serbia (2006-)
Turkey	Taiwan
Yemen	Tanzania
Zimbabwe	Turkmenistan

Organizations may be tied to ethnic groups through different mechanisms. For example, some parties have an explicit ethnic identity in their name, while others only have a manifested ethnic support base in elections. An organization can make explicit ethno-political demands or simply be composed of individuals from a particular ethnic group. Thus, the EPR-O dataset defines ethnic organizations as organizations that represent the interests of specific ethnic groups, in opposition to other ethnic groups in the country, (a) through explicit ethnic claims, (b) through recruitment along ethnic group lines, or (c) through electoral support along ethnic lines.

Ethnic claims are defined as public demands in favor of the rights or well-being of specific ethnic groups. Ethnic recruitment occurs when members overwhelmingly join, or are admitted to, the organization because they are from particular ethnic groups, while ethnic electoral support refers to voters’ overwhelmingly choosing a given political party over other parties because they are from specific ethnic groups. This broad definition allows us to detect ethnic organizations in distinct contexts, using different signals of their ethnic base. We use “because” in the definition to ensure that ethnic recruitment and support are not accidental (for example, a mere function of demographics), but a conscious decision by the individuals supporting/joining the organization.

We relied on both primary and secondary sources to identify organizations and determine their links to ethnic groups. Our “universe” of relevant political organizations is composed of entries in the following four sources: i) election archives of all national-level elections in the included countries between 1946 and 2013, ii) the Political Handbooks of the World (Banks et al. 2014), iii) the World Directory of Minorities and Indigenous Peoples (Minority Rights Group International 2007), and iv) the Cunningham (2013) self-determination movement (SDM) dataset. This covers a broad spectrum of possible organizational goals (governmental vs. territorial) and strategies (electoral and non-electoral; violent and non-violent). The full universe consisted of more than 2,600 political organizations. We then consulted a large number of sources, including original documents and websites of organizations, as well as scholarly texts, to decide whether a given organization could be associated with one or more ethnic groups as specified above. An organization was only linked to an ethnic group if at least one trustworthy source provided convincing evidence.

Organization-group linkages in EPR-O are many-to-many as each organization could be linked to more than one ethnic group, while an ethnic group may be represented by a single or several different organizations. For each organization-group link the dataset provides yearly codings of the stated goals for the group and the mobilization strategies employed to represent this group (e.g., use of violence). We focus on two broad claims by ethnic organizations: governmental power and territorial rights. Governmental power refers to aspirations to achieve power or influence for ethnic groups in the national government. Claims for territorial rights include demands for regional autonomy and/or secession. We code demands from explicit, public statements made by organizations and their leaders, as reported in the main primary and/or secondary sources. Figure A1 in Online Appendix III plots the relative frequency of these two broad claims at the organizational level over time.

Online Appendix II compares EPR-O to two alternative datasets on both violent and non-violent ethno-political organizations in terms of coverage, organizations, and key variables. For the purposes of this article, EPR-O has two main advantages. First, the more diverse set of countries included in the random sample should provide a better basis for generalizations than region-specific datasets, such as the Minorities at Risk Organizational Behavior dataset (MAROB) (Wilkenfeld, Asal, and Pate 2011). Second, EPR-O features a more comprehensive list of organizations than the Strategies of Resistance Data Project (SRDP), which is limited to states with self-determination movements (Cunningham, Dahl, and Frugé 2017).^6^ For instance, for Angola, EPR-O includes not only separatist organizations operating in Cabinda, but also violent and non-violent organizations focusing on the interests of other ethnic groups.^7^ This allows us to consider various dimensions when measuring the scope of ethnic group claims.

## Methodological Approach and Operationalization

### Estimation Strategy

Following the structure of our argument, our empirical analysis proceeds in two stages. We first test the relationship between movement fragmentation and the scope of ethnic group demands (hypothesis H1) using ordered logistic regression models. We then evaluate the effect of the scope of demands on ethnic civil conflict onset with logistic regression models at both the ethnic group (hypotheses H2a and H2b) and the organization levels (hypothesis H3). Our units of analysis are the ethnic-group year and the organization year, respectively. For the onset analysis, we use the King and Zeng (2001) rare events logit estimator and drop observations with ongoing civil conflicts. We account for temporal dependence within ethnic groups or organizations by a cubic polynomial of peace years (Carter and Signorino 2010). The ordered logistic regression models contain k − 1 lagged dummy variables (where k is the number of possible outcomes), each one referring to a particular value of the dependent variable in the preceding year.

With respect to conflict onset, organizations may make radical demands because they anticipate violence, and if so, we may overestimate the actual effect of the claims. Our main strategy to address this reverse causality concern is to explicitly model the scope of demands in the first part of our analysis. If we find evidence for a systematic effect of movement fragmentation on the scope of demands, even when controlling for prior conflict, we can be more confident that movement demands are not mere reflections of initial conflict proneness, but rather the result of movement-internal signaling and outbidding maneuvers, as suggested by our argument. Moreover, we use an additional indicator that records the scope of demands *in the first year* of an ethnic group’s political mobilization. The average group mobilization time before an ethnic civil conflict in our sample was over twenty years, long enough to make it implausible for claims to merely reflect expected future armed conflict.

### Measuring the Scope of Demands and Radicalization

**Figure 1.**
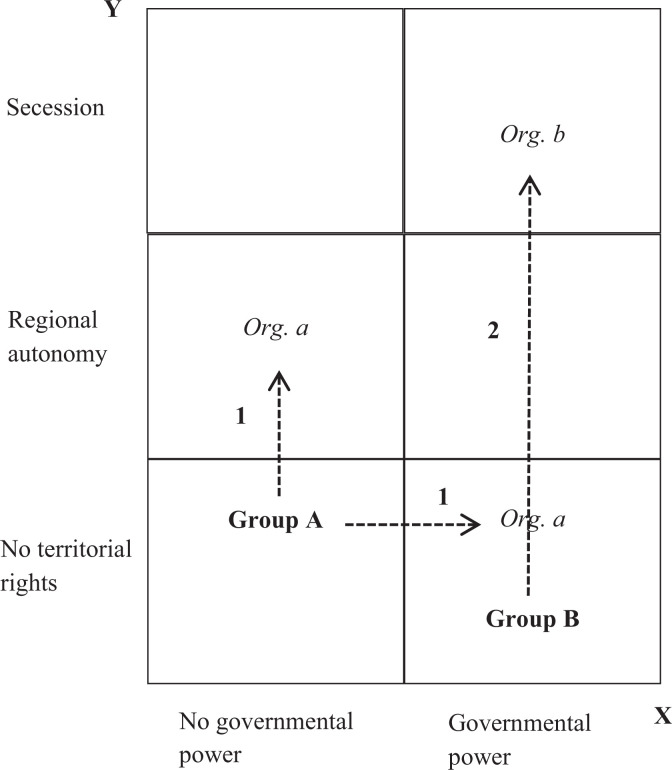
Calculating distance from status quo at the organizational level.

We operationalize the scope of demands as their distance from the status quo. We use the EPR-O dataset to determine the claims made by ethnic organizations regarding governmental power and territorial rights, and identify the status quo using information on ethnic groups’ access to national and regional-level executive power from the EPR dataset. We determine the scope of claims by locating both claims and status within a two-dimensional space of six fields and counting the distance in fields between the claims and the status quo (see Figure 1). The total distance value is given by the sum of horizontal and vertical moves necessary to get from the status quo to organizational demands. The distance is coded as 0 if no claims are made regarding governmental power and territorial rights.

On the governmental-power dimension (the horizontal axis in Figure 1), we focus on whether ethnic groups are included in national-level executive power or excluded, according to EPR. EPR codes ethnic groups as included if their political leaders occupy non-token positions of power in the relevant organs of executive power and exert influence on national policy (Vogt et al. 2015, 1331). The maximum distance of demands from the status quo is 1 on this dimension. This is the case when an organization representing an excluded ethnic group makes demands for access to national-level power. On the territorial-rights dimension, we distinguish between no territorial rights, regional autonomy within the existing state, and secession. In terms of the status quo, the EPR dataset codes ethnic groups as having regional autonomy if the state contains executive organs with decision-making power that operate below the state level (for example, departments) but above the local level *and* if group representatives exert actual influence within these organs (Vogt et al. 2015, 1331). The maximum distance of demands from the status quo is 2 on the territorial-rights dimension, which is the case when organizations demand separatism in the absence of any existing territorial rights.^8^

Figure 1 shows two hypothetical examples of the scope of demands within this two-dimensional space. Organization *a* representing ethnic group A demands access to governmental power as well as regional autonomy. Since group A currently is neither included in the central government nor in regional executives, these claims result in a scope value of 2, which results from a 1 on the governmental-power dimension and a 1 on the territorial-rights dimension. Organization *b* makes claims for secession on behalf of group B even though the group currently enjoys access to governmental power. This also results in a scope value of 2 on the territorial dimension.

Hypotheses H1, H2a and H2b refer to the ethnic-group level and the demands made by movements as a whole. Both the outbidding and the threat perception mechanisms in our argument should be mainly driven by the most radical organization within an ethnic movement. Thus, we use the maximum organizational value among all organizations representing an ethnic group in a given year as our movement-level indicator of the scope of demands. We also present robustness tests using the median value among all organizations, which should represent “average” movement demands. We combine the two dimensions into one single indicator, because from the perspective of the state government, the loss of power and/or institutional changes resulting from demands on more than one dimension (for example, providing regional autonomy to a group plus offering it access to central government power) will be perceived as cumulative. Accordingly, we view the distance from the status quo as cumulative. In additional robustness tests, we gauge the separate effects of the governmental power and territorial rights measures on ethnic civil conflict and use dummy variables for each value on the two dimensions.

Hypothesis H3 refers to the organization level and how the radicalization of individual organizations can trigger ethnic civil conflict. We define radicalization as an increase in the distance of demands from the status quo from one year to the next. Hence, we test hypothesis H3 using a dummy variable indicating if an individual organization increased its demands from one year to the next. In order to minimize the risk of endogeneity, we lag the variable by one year, thus capturing changes in the scope of demands from t_–2_ to t_−1_.

### Ethnic Civil Conflict Onset

The UCDP/PRIO Armed Conflicts Dataset defines conflicts as a contested incompatibility over government or territory between two parties, of which one is the government of a state and the other an organized non-state actor, resulting in at least twenty-five battle-related deaths per year (Gleditsch et al. 2002). We treat ethnic groups as experiencing a civil conflict if a rebel organization recruited fighters from a particular ethnic group *and* made public claims on behalf of the group, as coded in the ACD2EPR dataset (Wucherpfennig et al. 2012). Overall, the group-level sample contains seventy ethnic civil conflict onsets (about 0.6 percent of all ethnic-group years). At the organizational level, we identified the organizations in EPR-O that are listed as civil conflict actors in the ACD data and coded a civil conflict onset in the first year an organization appears in an ACD dyad. Overall, the sample contains ninety-eight organizational onsets of civil conflict (about 0.8 percent of all organization years).^9^

### Movement Fragmentation

Following previous studies (Cunningham 2013; Cunningham, Bakke, and Seymour 2012), we measure fragmentation by the logged number of EPR-O organizations that claim to represent the ethnic group. In the organization-level analysis, we use an indicator of the logged number of *other* organizations representing the same ethnic group as the organization in question.

### Control Variables

In the first stage of our analysis, we take into account a series of factors that could influence both movement fragmentation and the scope of claims. The first set refers to groups’ structural resources, and we include relative group size, the logged number of trans-border ethnic kin connections, and a dummy variable for whether a group is territorially concentrated from the EPR data. Moreover, we control for intra-ethnic linguistic and religious divisions, using two counts of the number of different linguistic and religious segments in an ethnic group from the EPR-ED data (Bormann, Cederman, and Vogt 2017). Intra-ethnic cleavages and the degree of territorial concentration can affect an ethnic group’s bargaining power and the scope of claims (Cetinyan 2002; Jenne, Saideman, and Lowe 2007) as well as the propensity for political fragmentation (Seymour, Bakke, and Cunningham 2016; Toft 2002; Weidmann 2009). Similarly, ethnic kin in neighboring states may increase material support (Jenne 2007), but transnational assistance can also foster intra-group divisions (Seymour, Bakke, and Cunningham 2016).^10^

The scope of demands made by ethnic groups obviously depends on the status quo itself, and groups’ political situation might also affect their propensity for fragmentation. Hence, our second set of controls refers to the political status quo of the ethnic group, measured by three variables: first, a political exclusion dummy, indicating whether the group lacks meaningful representation in government in a given year, according to EPR; second, a regional autonomy dummy variable denoting whether a group has meaningful representation in a sub-national executive organ; and finally, a dummy variable that records whether an ethnic group was downgraded in its EPR power status within the five years prior to the year in question to consider changes in the status quo.

The third set of controls captures the characteristics and actions of the government, which also affect both fragmentation and claim-making (Seymour, Bakke, and Cunningham 2016). We control for the degree of democracy and economic development as indicators of governments’ institutional and economic capacity to accommodate group demands, using V-Dem’s liberal democracy index (Coppedge et al. 2015) and a logged GDP per capita variable.

Previous studies suggest that the occurrence of violence increases the likelihood of fragmentation (Asal, Brown, and Dalton 2012; Seymour, Bakke, and Cunningham 2016), and such violence could also bolster the demands of movements. Hence, our fourth set of control variables captures earlier instances of political violence within a given government-movement dyad. First, we include the number of previous civil conflicts involving an ethnic group, based on the ACD2EPR dataset. Second, we include a dummy variable that indicates whether a given ethnic movement (or a given organization in the organization-level analysis) used violence against the government below the threshold for civil conflict in the foregoing year, based on the corresponding variable from the EPR-O dataset. At the organizational level, we additionally control for whether the ethnic group as a whole was involved in a civil conflict in the foregoing year.

Since both movement fragmentation and group claims might be influenced by the strategic environment, our fifth set of controls takes into account other potential challengers in a given country (Walter 2006). We control for both the logged number of other politically relevant ethnic groups in the country, according to the EPR dataset, and the *demands made* by these groups. The latter variable indicates for each group year the average value of the scope of the demands made by all other groups in the country. This should also help us distinguish the demands of a specific movement from underlying country-level factors that may influence ethno-political claim-making more generally. In addition, we consider a country’s logged population size. Sixth, existing research highlights factionalized leadership as a source of fragmentation (Asal, Brown, and Dalton 2012). While we cannot directly measure movement-internal personal rivalries or strategic disagreements, we control for the duration of mobilization (i.e. the number of years since the establishment of a group’s first political organization), assuming that internal rivalries and competition should become more likely over time.

In the second stage of our analysis we also control for structural group resources, existing inter-group inequality, the degree of democracy and economic capacity, previous instances of political violence, the strategic environment, as well as mobilization duration as these factors can all be expected to affect the likelihood of ethnic civil conflict (Cederman, Gleditsch, and Buhaug 2013; Gurr 1994; Jenne 2007; Walter 2006). In addition, at the organizational level, the strength of an organization could influence both radicalization and the risk of violent escalation. Following previous studies, we consider the organization’s age as a proxy of its institutional capacity (Asal and Rethemeyer 2008; Horowitz 2010). Finally, all models control for time trends using a calendar year variable. Tables A1 and A2 in Online Appendix III provide summary statistics of the main independent variables at both the group and organization levels. All right-hand side variables are lagged by one year in the statistical analysis.

## Empirical Results

### Movement Fragmentation and the Scope of Demands

We start our analysis with the relationship between internal fragmentation and movement demands. Table 2 summarizes the regression results. We begin with a parsimonious model that contains the fragmentation variable and three basic indicators of groups’ structural resources. The effect of the logged number of organizations is positive and statistically significant. This result does not change when we add the rest of our control variables in Model 2. Model 3 restricts the sample to ethnic group years with at least one political organization recorded in our dataset to ensure that the observed effect of fragmentation on the scope of demands is not simply a byproduct of mobilization itself. The effect of the logged number of organizations becomes even somewhat stronger in this model. In substantive terms, the predicted probability of an increase in the scope of demands from 0 to 1, from 1 to 2, and from 2 to 3 from one year to the next increases by about 17 percent, 27 percent, and 11 percent, respectively, when moving the fragmentation variable from its minimum to its maximum value.^11^

**Table 2.** Movement Fragmentation and the Scope of Demands. Regression Results.

	Model 1	Model 2	Model 3
N organizations (logged)	.12**	.12*	.14*
	(.04)	(.05)	(.06)
Relative group size	−.46*	.43	−.01
	(.23)	(.43)	(.58)
N of TEK connections (logged)	.11	.04	.07
	(.07)	(.10)	(.13)
Geographic concentration	.17	.77**	.79**
	(.24)	(.28)	(.27)
Excluded		1.52***	1.85***
		(.22)	(.35)
Regional autonomy		−1.01***	−1.70***
		(.28)	(.39)
Downgraded in last five years		1.34***	1.78***
		(.20)	(.29)
Liberal democracy		.80	.00
		(.41)	(.42)
GDP per capita		−.00	.13
(logged)		(.09)	(.09)
N of years of mobilization		.02*	.02*
		(.01)	(.01)
Use of small-scale violence by movement		−.36	−.27
		(.20)	(.26)
Group’s conflict history		.24	.20
		(.13)	(.14)
N of other groups in country (logged)		−.05	.17
		(.10)	(.14)
Scope of demands of other groups		.20	−.10
		(.17)	(.21)
Country population (logged)		.02	.13
		(.09)	(.08)
Calendar year		−.01	−.02**
		(.01)	(.01)
N of religious segments		.14	.21
		(.10)	(.14)
N of linguistic segments		−.12	−.24
		(.12)	(.16)
k − 1 lagged outcome dummy variables	Yes	Yes	Yes
Cut 1	5.27***	−5.00	−21.12
	(.41)	(13.11)	(11.75)
Cut 2	12.05***	2.33	−12.58
	(.62)	(12.86)	(11.47)
Cut 3	19.22***	10.08	−4.19
	(.80)	(13.06)	(11.60)
N	12,403	12,299	5,060
Log likelihood	−1,062.50***	−988.21***	−642.53***

*Note*: Robust standard errors, with clustering on countries, in parentheses. *p < 0.05, **p < 0.01, ***p < 0.001.

Our sample includes a number of cases that aptly illustrate how internal fragmentation and the resulting inter-organizational competition can lead to increased demands on state governments. For example, the National Awami Party/National People’s Party (NAP), a leading Bengali organization in 1960s Pakistan mostly composed of former Awami League members, first embraced a leftist-oriented, multiethnic stance toward Bengali nationalism. It then shifted toward a more ethnically based approach in its 1965 election manifesto, where it advanced its first claim for full regional autonomy for East Pakistan. In turn, the Awami League issued its own Six-Points Programme in 1966, which propagated a much more far-reaching vision of a quasi-independent East Pakistan with the rights to issue its own currency, collect its own taxes, and establish its own militia (Leonard 2006). Similarly, competition over leadership within the Tatar movement between the Tatar Public Center (TOTs, TPC), on one side, and the Ittifak Party and its Azatlyk youth organization, on the other, contributed to increasingly greater demands for autonomy from Russia at the beginning of the 1990s (Tanrisever 2002, 191-92).

Apart from movement fragmentation, the only other variables that have a relatively consistent effect on the scope of ethnic group demands in these models are territorial concentration, mobilization duration, and the three variables capturing a group’s political status quo. Unsurprisingly, the longer mobilization lasts, the more likely fragmentation becomes, presumably at least partly as a result of personal competition over leadership (Asal, Brown, and Dalton 2012). Also, politically marginalized groups are more likely to make demands that are further from the status quo. This suggests that, apart from the collective grievances that can be mobilized by movement leaders, another mechanism leading from inter-group inequality to violent conflict escalation might be the tendency of marginalized groups to make demands that appear radical to the ruling elite, thus increasing the risk of bargaining breakdown. Finally, the positive effect of territorial concentration on the scope of movement demands confirms the importance of group resources for claim-making (Cetinyan 2002; Jenne, Saideman, and Lowe 2007). We find no systematic effect of prior instances of political violence on the scope of movement demands, suggesting that movement demands are unlikely to be mere reflections of the underlying conflict proneness of a given state-movement relationship.

Online Appendix IV presents a series of robustness tests, including the use of fixed effects to neutralize unobserved heterogeneity at the movement level, as well as dynamic indicators of radicalization and fragmentation to address concerns of reverse causality. Moreover, our results using the median, rather than maximum, organizational scope value mirror those in Table 2. Thus, internal fragmentation seems to shift ethnic movements as a whole toward more radical claims, rather than simply producing radical outliers. Overall, our results lend support to hypothesis H1 and the first stage of our argument.

### From Claims to Violence: Ethnic Civil Conflict Onset

Our conflict onset analysis first tests the direct effect of the scope indicator on civil conflict outbreak at the ethnic group level (hypothesis H2a). We begin again with the most parsimonious model. The results of Model 4 in Table 3 show that the scope of demands has a positive and statistically significant effect, indicating a higher risk of ethnic civil conflict the further the distance between demands and the status quo. Model 5 adds the rest of our control variables. The coefficient of the scope indicator decreases, but remains robustly related to the risk of ethnic civil conflict onset. This finding does not change when we add country and year-fixed effects in Model 6, suggesting that the result is unlikely to be driven by unobserved heterogeneity across countries and time.

**Table 3.** The Scope of Demands, Organizational Radicalization, and Ethnic Civil Conflict Onset. Regression Results.

	Group Level				Organization Level	
	Model 4	Model 5	Model 6	Model 7	Model 8	Model 9
Scope of demands	.73***	.49**	.56**	.51*		
	(.17)	(.18)	(.21)	(.26)		
Scope of demands * N of other groups in country				−.00		
				(.15)		
Radicalization of organization					1.21*	2.85**
					(.59)	(1.01)
N organizations (logged)	.36***	−.01	−.23	−.01		
	(.09)	(.10)	(.12)	(.10)		
N other organizations in the same movement (logged)					.01	.07
					(.26)	(.26)
Relative group size	.13	.75	.69	.76	.96	1.00
	(.83)	(.63)	(.72)	(.61)	(1.01)	(.78)
N of TEK connections (logged)	.28*	.38**	.78***	.38**	−.35***	−.35
	(.13)	(.12)	(.21)	(.12)	(.09)	(.25)
Geographic concentration	.69	.11	.85	.11	−.14	.02
	(.68)	(.77)	(.55)	(.76)	(.61)	(.90)
Excluded		.84*	1.13**	.84*	1.05*	1.17*
		(.38)	(.43)	(.39)	(.48)	(.54)
Regional autonomy		.62	.91*	.62	.55	1.49**
		(.33)	(.40)	(.33)	(.40)	(.48)
Downgraded in last five years		2.05***	2.05***	2.04***	.62	.33
		(.51)	(.50)	(.51)	(.35)	(.39)
Liberal democracy		−.12	1.43	−.12	−.79	.63
		(1.13)	(1.42)	(1.13)	(.85)	(2.05)
GDP per capita (logged)		−.13	−.44	−.13	.09	.30
		(.17)	(.41)	(.18)	(.08)	(.51)
N of years of mobilization		.03**	.05***	.03**		
		(.01)	(.01)	(.01)		
Organization age					.03***	.03***
					(.00)	(.01)
Use of small-scale violence by movement/organization		1.99***	2.21***	1.99***	2.46***	2.52***
		(.60)	(.63)	(.58)	(.47)	(.53)
Occurrence of civil conflict at group level					−.98***	−1.10***
					(.18)	(.29)
Group’s conflict history		−.13	−.22	−.14	.19	.09
		(.17)	(.26)	(.17)	(.10)	(.16)
N of other groups in country (logged)		.02	1.42	.02	−.47	−.85
		(.18)	(1.03)	(.20)	(.30)	(1.46)
Scope of demands of other groups		−.16	−1.16*	−.17	.26	−.03
		(.23)	(.58)	(.23)	(.25)	(.42)
Country population (logged)		−.14	−.50	−.14	.06	.28
		(.11)	(.70)	(.11)	(.24)	(1.39)
Calendar year		.00		.00	−.01	
		(.01)		(.01)	(.01)	
Cubic polynomial of peace years	Yes	Yes	Yes	Yes	Yes	Yes
Constant	−4.32***	−3.46	1.85	−3.33	10.07	−13.29
	(.54)	(23.82)	(13.72)	(23.56)	(13.04)	(23.40)
Country-fixed effects	No	No	Yes	No	No	Yes
Year-fixed effects	No	No	Yes	No	No	Yes
N	11,825	11,723	4,930	11,723	11,967	5,349
Log likelihood	−355.85***	−299.64***	−214.06***	−299.63***	−345.95***	−287.78***

Note: Standard errors in parentheses. Clustering on countries in Models 4, 5, 7, 8, and on ethnic groups in Models 6 and 9. Log-likelihood figures obtained from standard logistic regressions. *p < 0.05, ** *p* < 0.01, ***p < 0.001.

**Figure 2.**
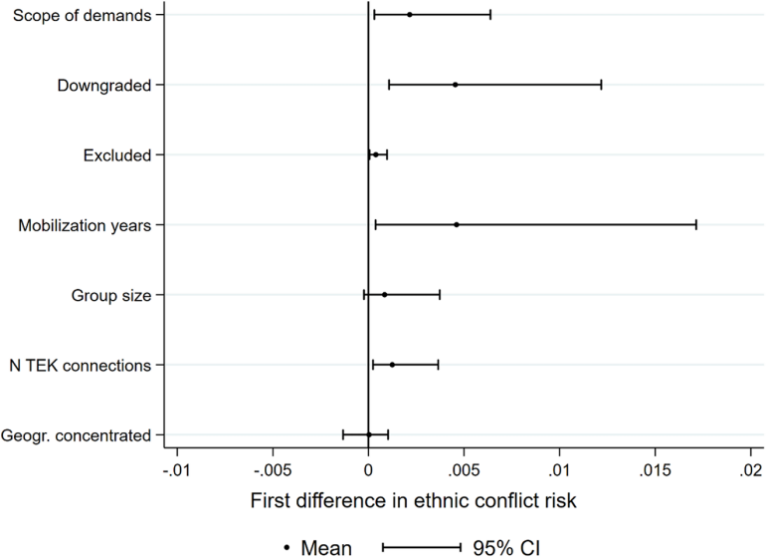
Substantive effects. Notes: Based on Model 5 in Table 3 and calculated with simulation methods using Clarify (King, Tomz, and Wittenberg 2000). Graph shows first differences in the predicted ethnic civil conflict risk when moving any of the independent variables listed on the y-axis from their minimum to their maximum values. All other variables held constant at their mean, median, or mode.

Figure 2 contextualizes the substantive effect of the scope indicator by comparing it to that of other explanatory variables. Based on Model 5, the figure displays the first differences in the predicted probability of conflict onset when moving any of these variables from their minimum to their maximum value, while holding all other variables constant at their mean, median, or mode. The associated increase in the risk of ethnic civil conflict onset is relatively small (due to the low overall conflict risk in the sample), but the scope measure has a larger effect than, for example, political exclusion and the number of trans-border ethnic kin connections. Overall, these results lend strong support to hypothesis H2a, suggesting that there is a direct effect of the scope of movement demands on the risk of violent conflict escalation.

Model 7 tests the second mechanism leading from movement demands to civil conflict outbreak, which runs through the government’s fear of setting precedents. Since this effect of demands is conditional on potential future imitators, we include an interaction term of our scope indicator with the logged number of other ethnic groups in the country. Otherwise, the model is identical to Model 5. The results do not support hypothesis H2b, as the interaction term remains insignificant. This suggests that the effect of radical demands does not depend on the existence of potential future imitators, but results from an escalation in the dyadic government-movement interaction.

Beyond the demands made by ethnic movements, we find that politically excluded groups and those whose political status was downgraded within the five foregoing years have a significantly higher probability of ethnic civil conflict. Hence, while the focus on the political demands advanced by ethnic movements allows us to get closer to the causal mechanisms of conflict escalation, our results also uphold earlier findings that emphasize the importance of group-level indicators of horizontal inequality (Cederman, Gleditsch, and Buhaug 2013; Gurr 1994; Stewart 2008). In addition, support for a movement from ethnic kin in other states makes an armed confrontation with the government more likely. This indicates that group resources matter for the bargaining between states and ethnic movements and, by extension, the risk of civil conflict outbreak (e.g., Cetinyan 2002; Jenne, Saideman, and Lowe 2007).

Interestingly, the count of the number of organizations within an ethnic movement is only statistically significant in one of these models. Together with the results from the first stage of our analysis, this suggests that the effect of fragmentation on armed conflict found in previous studies (e.g., Cunningham 2013) works in part through claim-making. Finally, we do not find any significant positive effect of the scope of demands made by *other* groups in the country on the risk of ethnic civil conflict. This indicates that the effect of our main explanatory variable is specific to the group that advances such demands and not an artifact of underlying factors that influence ethnic politics in a given country more generally.

Online Appendix V provides additional robustness tests. The results reveal that the effect of the scope of demands pertains to both the governmental and territorial-rights dimensions, which suggests that the risk of violent escalation is not confined to conflicts over territory, as emphasized in previous studies (Cunningham, Bakke, and Seymour 2012; Toft 2002; Walter 2006). Moreover, when using the first-year scope indicator, the results confirm the direct effect of radical demands on ethnic civil conflict outbreak: the larger the distance between the demands and the status quo in the first year of group mobilization, the higher the risk of subsequent conflict outbreak.

This also points to two different scenarios of escalation in the dyadic government-movement interaction (see Table A5 in Online Appendix V for examples). Some ethnic movements advance their maximum demands at the very beginning of the mobilization process. For example, the Karen National Union advanced secessionist claims on behalf of the Kayin/Karen group in Myanmar from its foundation in the latter half of the 1940s. The demands were met with intransigence by the newly independent Burmese state, and latent tensions quickly escalated into outright war. In Zimbabwe, ZAPU (and later ZANU) aimed at overturning White minority rule from the outset. In response, the Southern Rhodesian government banned the organization and suppressed any public campaigns, ultimately forcing the African nationalist movement to pursue its goals by violent means. This example also highlights how claims made by marginalized groups that appear radical to the ruling elite (and thus provoke a backlash) can play a crucial role in the escalation process leading from inter-group inequality to civil conflict outbreak.

In other cases, movements increase their demands over the course of mobilization, which exacerbates tensions and may ultimately lead to outright armed confrontation. This was the case in the aforementioned example of Bengali mobilization in Pakistan. Following the presentation of its 1966 Six-Points Programme, the Awami League won a clear majority in East Pakistan in the 1970 elections. Threatened by the prospects of East Pakistani separatism, Pakistan’s military intervened in the coalition talks and cracked down on Bengali mobilization. This only served to further increase grievances in East Pakistan, and the Awami League responded to the army’s brutal repression by calling for a noncooperation movement, ultimately leading to civil war and independence (Leonard 2006).

To evaluate the impact of such radicalization processes in more detail, we now move to the organization-level analysis. Hypothesis H3 holds that as individual organizations increase their demands, they should be more likely to become engaged in civil conflict. The results of Model 8 in Table 3 lend support to this hypothesis. The dummy variable capturing organization-specific radicalization exerts a positive and statistically significant effect on the risk of ethnic civil conflict onset. Thus, our results provide evidence for a direct effect of radicalization on ethnic civil conflict onset: increasingly radical demands tend to push organizations toward violent action. This finding remains robust when we add country and year-fixed effects in Model 9.

In addition, the results show that older organizations are more likely to become involved in civil violence. Given that age tends to be associated with higher institutional capacity, this finding again underlines the important role of opposition resources. Unsurprisingly, the use of small-scale violence against the government either at the movement or the organization level increases the likelihood of civil conflict. By contrast, an individual organization is less likely to take up arms against the government if the group it represents was already engaged in an ethnic civil conflict in the foregoing year. This mirrors recent evidence that individual organizations have an incentive to diversify their strategies compared to movement-internal competitors (Cunningham, Dahl, and Frugé 2017). Importantly, the robust effect of our radicalization indicator implies that increasingly radical demands advanced by specific organizations have an independent impact over and beyond the effect of prior instances of violence within the government-movement dyad. In short, our results suggest that advancing increasingly radical demands locks ethno-political organizations into a spiral of escalation that increases the risk of armed civil conflict.

## Conclusions

Almost three decades ago, Diehl (1992) criticized international conflict research for disregarding the content of disputes. The same observation applies to current civil war research. Recent studies of civil violence have advanced our knowledge of actor fragmentation, power relations, and alliances in conflict dynamics and the role of ideology in armed group behavior. Yet, the neglect of conflict actors’ claims in quantitative research has left us without systematic evidence on many important mechanisms of conflict escalation highlighted by existing theories and case studies. We have argued that in addition to genuine group aspirations, demands advanced on behalf of ethnic groups can serve as organization-external and internal signaling devices. Especially in the context of fragmented movements, individual organizations and their leaders have incentives to use radical demands to signal strength to the state, boost intra-organizational cohesion, and outbid movement-internal rivals – with important negative externalities for the bargaining process with the government. On the one hand, far-reaching demands by opposition movements likely affect governments’ threat perception. On the other hand, organizations that make increasingly radical demands lock themselves into a spiral of escalation, afraid of paying the reputation costs from backing down.

We find empirical support for a strong positive relationship between movement fragmentation and the scope of ethnic group demands, as well as a direct effect of the latter and of organization-specific radicalization on the risk of civil violence. Political demands are always the result of environmental circumstances and thus inherently endogenous, but our empirical results point to an independent effect of the scope of demands. While we cannot definitively rule out possible reverse causation, this concern is mitigated by the fact that far-reaching demands at the outset of groups’ mobilization still have a clear impact on the risk of civil conflict many years later. We also show that prior ethnic civil conflict does not systematically increase the scope of movement demands, suggesting that the latter is not just a function of the underlying conflict proneness of a given state-movement relationship. Although the EPR-O dataset is currently limited to forty countries, the random sampling approach makes it unlikely that the findings suffer from systematic biases, and if anything the limited number of observations raises the bar for finding statistically significant results.

Overall, our results support our claim that radical demands reflect deliberate choices made by political organizations and their leaders. This is in line with actor-centered arguments from studies of social movements, which emphasize the importance of political agency above and beyond structural conditions (e.g., Sharp 2005; Stephan and Chenoweth 2008). Our findings also contribute to recent studies of ethnic grievances and civil conflict, which have mostly focused on measuring objective horizontal inequality, rather than the concrete demands that ethnic organizations advance in response to such inequality (e.g., Cederman, Wimmer, and Min 2010; Stewart 2008; Vogt 2019). By confirming the relevance of claims in ethnic outbidding (Horowitz 1985, 185-228; Kaufman 1996; Rabushka and Shepsle 1972), we provide new insights into the complex processes of escalation from objective inequality to the outbreak of ethnic civil conflict. Contrary to extant research on issue indivisibility and conflict escalation (e.g., Hassner 2009; Svensson 2007; Toft 2007), we have shown that, independent of the particular dimension of political claim-making, demands that are further away from the status quo are likely to be perceived as more radical and, thus, to increase the risk of violent conflict escalation.

Hence, our study also feeds into the long-standing debate on the political consequences of radicalism, which has typically focused on the achievement of movement goals (e.g., Gamson 1975; Haines 1995; McCammon, Bergner, and Arch 2015). Applying a new measure of radical demands to a global sample of a particular type of movements, we show that in the context of ethno-political mobilization, the negative social externalities are likely to outweigh potential organization or movement-level gains in the form of funding or political success. Yet, organizations could also attempt to raise their own profile through moderation, that is, by “underbidding” their movement-internal rivals’ demands. For instance, social movement scholars have shown that “radical flanks” often only emerge as such once other factions strategically downscale their demands to secure individual benefits from the state and third parties (e.g., McCammon, Bergner, and Arch 2015). Thus, one promising avenue for future research using the new EPR-Organizations dataset would be a systematic analysis of the causes and consequences of de-escalation in terms of movement demands: why and when individual organizations downscale their claims and how such moderation affects the strategic interactions with the state government and the risk of armed conflict.

Finally, our approach could also inform research on radicalization in the field of terrorism studies (e.g., McCauley and Moskalenko 2017; Moghaddam 2005). In line with previous works in this field (Abrahms 2012; Asal and Rethemeyer 2008; Piazza 2009), we find that the publically declared goals of organizations have an important impact on conflict dynamics. This implies that the process of radicalization can be conceptually captured partly by increases in the scope of political goals. Recent psychological research argues that “radicalization of opinion” and “radicalization of action” may be separate processes for individuals (McCauley and Moskalenko 2017). The results of our study suggest that outbidding is a crucial mechanism at the organizational level that leads from one to the other.

## Supplemental Material

Supplemental Material, sj-do-1-jcr-10.1177_0022002721996436 - From Claims to Violence: Signaling, Outbidding, and Escalation in Ethnic Conflict Click here for additional data file. Supplemental Material, sj-do-1-jcr-10.1177_0022002721996436 for From Claims to Violence: Signaling, Outbidding, and Escalation in Ethnic Conflict by Manuel Vogt, Kristian Skrede Gleditsch and Lars-Erik Cederman in Journal of Conflict Resolution

Supplemental Material, sj-dta-1-jcr-10.1177_0022002721996436 - From Claims to Violence: Signaling, Outbidding, and Escalation in Ethnic Conflict Click here for additional data file. Supplemental Material, sj-dta-1-jcr-10.1177_0022002721996436 for From Claims to Violence: Signaling, Outbidding, and Escalation in Ethnic Conflict by Manuel Vogt, Kristian Skrede Gleditsch and Lars-Erik Cederman in Journal of Conflict Resolution

Supplemental Material, sj-dta-2-jcr-10.1177_0022002721996436 - From Claims to Violence: Signaling, Outbidding, and Escalation in Ethnic Conflict Click here for additional data file. Supplemental Material, sj-dta-2-jcr-10.1177_0022002721996436 for From Claims to Violence: Signaling, Outbidding, and Escalation in Ethnic Conflict by Manuel Vogt, Kristian Skrede Gleditsch and Lars-Erik Cederman in Journal of Conflict Resolution

Supplemental Material, sj-dta-3-jcr-10.1177_0022002721996436 - From Claims to Violence: Signaling, Outbidding, and Escalation in Ethnic Conflict Click here for additional data file. Supplemental Material, sj-dta-3-jcr-10.1177_0022002721996436 for From Claims to Violence: Signaling, Outbidding, and Escalation in Ethnic Conflict by Manuel Vogt, Kristian Skrede Gleditsch and Lars-Erik Cederman in Journal of Conflict Resolution

Supplemental Material, sj-pdf-1-jcr-10.1177_0022002721996436 - From Claims to Violence: Signaling, Outbidding, and Escalation in Ethnic Conflict Click here for additional data file. Supplemental Material, sj-pdf-1-jcr-10.1177_0022002721996436 for From Claims to Violence: Signaling, Outbidding, and Escalation in Ethnic Conflict by Manuel Vogt, Kristian Skrede Gleditsch and Lars-Erik Cederman in Journal of Conflict Resolution

## Notes

1. Walter (2017) proposes a relational approach to measuring extremism, but her discussion remains focused on religion. Zuber and Szöcsik (2015) measure the scope of demands by ethnonational minority parties in Europe relative to the party system average.

2. Walter (2017) makes a similar argument with respect to religious extremism.

3. The fragmentation of ethnic movements is in itself an important political outcome that has been analyzed in previous studies (e.g., Asal, Brown, and Dalton 2012; Seymour, Bakke, and Cunningham 2016). Given that the sources of fragmentation might also affect the scope of movements’ claims, we return to this issue in the control variables section below.

4. In principle, organizations could also attempt to distinguish themselves from movement-internal competitors through moderation. We will return to this issue in the conclusion.

5. The sample is drawn from the universe of countries in the EPR dataset.

6. The SRDP is based on Cunningham’s (2013) SDM dataset, which records self-determination organizations in 24 out of the 40 countries included in EPR-O. In these twenty-four states, EPR-O features an average of 22.6 organizations per country, compared to an average of 15.1 in the SDM dataset.

7. Figure A1 in Online Appendix III also shows that in the majority of years, less than 50 percent of all organizations contained in EPR-O made claims relating to territorial rights.

8. Note that organizations can also simultaneously make claims for both secession and access to national-level power in the existing state. For example, the latter can be seen as a short-term goal, with the long-term objective of attaining an independent state. Or organizations might aim to achieve influence in the government in order to destabilize existing state institutions from within (similar to anti-system parties) in the hope that this facilitates their secessionist attempts.

9. The difference in the number of conflict onsets stems from the fact that a civil conflict may involve two or more rebel organizations.

10. While it would be preferable to measure external (military) support more directly, the only such global data we are aware of stem from the MAR project (Minorities at Risk Project 2009). Yet, they only cover part of the ethnic groups included in our sample. Thus, following the existing literature (e.g., Saideman 1997), we use trans-border ethnic kin connections as an indicator of the external support *potential*.

11. The predicted probabilities were calculated with simulation methods using Clarify (King, Tomz, and Wittenberg 2000). All other variables were held constant at their mean, median, or mode.
