# Supplementary material for: From Claims to Violence: Signaling, Outbidding, and Escalation in Ethnic Conflict
Source: J Conflict Resolut. 2021 Mar 2;65(7-8):1278–307. doi: 10.1177/0022002721996436 (PMC8358586; doi:10.1177/0022002721996436)
Supplement: Supplemental Material, sj-pdf-1-jcr-10.1177_0022002721996436 - From Claims to Violence: Signaling, Outbidding, and Escalation in Ethnic Conflict [file sj-pdf-1-jcr-10.1177_0022002721996436.pdf]

Appendix I: EPR-O Organizations by Country and Ethnic Group

Country	Ethnic group	Organizations
Algeria	Arabs	Front de Libération Nationale (FLN)
Algeria	Berbers	Berber Cultural Movement (MCB); Coordinations of Kabylia (CADC); Front des Forces Socialistes (FFS); Movement for the autonomy of Kabylie (MAK); Rassemblement pour la Culture et la Démocratie (RCD)
Angola	Bakongo	Frente Nacional de Libertacao de Angola (FNLA); Partido Democratico para o Progresso de Alianca Nacional Angolana (PDP-ANA)
Angola	Cabindan Mayombe	Comando Militar de Libertacao de Cabinda (CMLC); Comité Comunista de Cabinda (CCC) / Communist Committee of Cabinda; FLEC-Conselho Superior Alargado (FLEC-CSA); FLEC-Forcas Armadas Cabindesas (FLEC/FAC); FLEC-Renovada; Forum Cabindes para o Dialogo (FCD); Frente Democratica de Cabinda (FDC); Frente para a Libertacao do Enclave de Cabinda / Forcas Armadas de Libertacao Cabinda (FLEC/FALC); Movimento Popular de Libertacao de Cabinda (MPLC); Movimiento para a Libertacao de Cabinda (MOLICA); Mpalabanda - Associacao Civica de Cabinda (MACC); Uniao Nacional de Libertacao de Cabinda (UNLC)
Angola	Lunda-Chokwe	Partido de Renovacao Social (PRS)
Angola	Mbundu-Mestico	Movimento Popular de Libertacao de Angola (MPLA)
Angola	Ovimbundu-Ovambo	Uniao Nacional da Independencia Total de Angola (UNITA)
Australia	Aborigines	Aboriginal Advancement League of Victoria; Aboriginal Provisional Government; Australia's Indigenous Peoples' Party; Australians for Native Title and Reconciliation (ANTAR); Indigenous Law Centre, University of New South Wales, Faculty of Law
Australia	Whites	Australia First Party; Australian League of Rights; Pauline Hanson's One Nation Party
Azerbaijan	Armenians	Republic of Nagorno-Karabakh / Republic of Mountainous Karabakh
Azerbaijan	Azeri	Karabakh Liberation Army / Karabakh Liberation Organization
Azerbaijan	Lezgins	Lezgin Democratic Party / Lezgin Democratic Party of Azerbaijan; Lezgin National Council (LNC); Sadval (original) / Lezgin National Movement
Bangladesh	Bengali Muslims	BJI / JIB – Jamaat-e-Islami Bangladesh (Islam Conference Bangladesh); BML – Bangladesh Moslem League; IOJ – Islami Oikya Jote (Islamic Unity Front); JMB - Jamaat-ul-Mujahidin Bangladesh; JMJB - Jagrata Muslim Janata Bangladesh; Kaderia Bahini; Parbattya Gono Parishad (Hill Tracts People’s Council); Sachetan Islami Janata; Shahadat al-Hikma
Bangladesh	Biharis (Urdu-Speaker)	BJI / JIB – Jamaat-e-Islami Bangladesh (Islam Conference Bangladesh); BML – Bangladesh Moslem League; IOJ – Islami Oikya Jote (Islamic Unity Front)
Bangladesh	Tribal-Buddhists	Hill Watch Human Rights Forum; Mukti Parishad; NFC - National Freedom Council; PCJSS - Parbattya Chattagram Jana Sanghati Samiti (Chittagong Hill Tribal People's Coordination Association); PCP - Pahari Chhatra Parishad / Prashit Sanchay (Hill Student's council); Shanti Bahini; UPDF - United Peoples' Democratic Front; Women's Hill Federation
Belgium	Flemings	Christen-Democratisch en Vlaams (CD&V); Groen (Green); Libertair, Direct, Democratisch (LDD); Meervoud VZW; Nieuw-Vlaamse Alliantie (N-VA); Open Vlaamse Liberalen en Democraten (Open VLD); Overlegcentrum van Vlaamse

Country	Ethnic group	Organizations
		Verenigingen; Partij voor Vrijheid en Vooruitgang, Vlaanderen (PVV, Party of Freedom and Progress, Flanders); Radicale Omvormers en Sociale Strijders voor een Eerlijker Maatschappij (ROSSEM); Sociaal-Liberale Partij (SLP - Spirit); Socialistische Partij Anders (PSA); Vlaams Belang (VB); Vlaamse Concentratie (VC); Vlaamse Militanten Orde (VMO); Vlaamse Volksbeweging; Volksunie (VU); Voor Individuele Vrijheid en Arbeid in een NieuweToekomst (VIVANT)
Belgium	Germans	Frauenliga/Vie Féminine VoG
Belgium	Walloon	Centre Démocrate Humaniste (CDH); Front National (FN); Front Wallon pour l'Unité et la Liberté de la Wallonie (FW); Fédéralistes Démocrates Francophones (FDF); Institut Jules Destrée; Parti Libéral (PL, Liberal Party); Parti des Réformes et de la Liberté en Wallonie (PRLW, Party of Reform and Freedom in Wallonia); Parti Populaire - Personenpartij (PP); Parti Réformateur Libéral (PRL); Parti Socialiste, Wallonia (PS, Wallonia); Parti Wallon des Travailleurs (PWT); Rassemblement Wallon (RW); Écologistes Confédérés pour l'organisation de luttes originales (Ecolo)
Bosnia and Herzegovina	Bosniaks/Muslims	Activist Democratic party / Party of Democratic Activity (A-SDA); Bosnian Patriotic Party / Bosnian Herzegovinic Patriotic Party (BPS) / BPS Sefer Halilovic; Coalition for Unity and Democratic Bosnia and Herzegovina (KCD BiH); Party for Democratic Action (SDA); Party of Bosnia-Herzegovina (SBiH); Union for a Better Future of Bosnia and Herzegovina / Alliance for a Better Future of BiH (SB BiH)
Bosnia and Herzegovina	Croats	Coalition New Croat Initiative and Croatian Christian Democratic Union (NHI-HKDU); Croat National Union / Croat National Alliance (HNZ); Croatian Defense Council (HVO) / Army of the Croatian Bosina and Hercegovina / Croatian Army of Defence; Croatian Defense Forces (HOS) / Croatian Defense Union / Croatian Defense Association; Croatian Democratic Union (HDZ); Croatian Democratic Union 1990 / HDZ 1990 / Croatians Together; Croatian Party of Rights (HSP); Croatian Peasants Party; New Croat Initiative (NHI)
Bosnia and Herzegovina	Roma	Institution for the Protection of Human Rights (Independent)
Bosnia and Herzegovina	Serbs	Alliance of Independent Social Democrats (SNSD); Allliance for Peace and Progress / People's Union for Peace; Coalition Together for Serbia / Together for Srpska (KZS); Democratic Patriotic Bloc (DPB); Party of Democratic Process (PDP); Radical Party of Republika Srpska (RS RS); Serb Democratic Party (SDS); Serbian Democratic Movement (DPS); Serbian National Alliance / Serb People's Alliance of the Republika Srpska (SNS / SNS RS); Serbian Radical Party of Republika Srpska (SRS RS); Socialist Party of Republika Srpska (SPRS) / Socialist Party
Botswana	Birwa	RETENG: The multi-cultural Coalition of Botswana
Botswana	Herero/Mbanderu	RETENG: The multi-cultural Coalition of Botswana
Botswana	Kalanga	Botswana People's Party (BPP); RETENG: The multi-cultural Coalition of Botswana; The Society for the Promotion of Ikalanga Language (SPILL)
Botswana	Kgalagadi	RETENG: The multi-cultural Coalition of Botswana
Botswana	Mbukushu	RETENG: The multi-cultural Coalition of Botswana
Botswana	San	Bokamoso Trust; D’Kar Trust / Kuru D’Kar Trust; Kgeikani Kweni / First People of the Kalahari (FPK); RETENG: The multi-cultural Coalition of Botswana
Botswana	Tswapong	RETENG: The multi-cultural Coalition of Botswana

Country	Ethnic group	Organizations
Botswana	Yeyi	Kamanakao Association; RETENG: The multi-cultural Coalition of Botswana
Brazil	Afrobrazilians	Centro de Estudos das Relações de Trabalho e as Desigualdades (CEERT); Coordenação Nacional de Entidades Negras (CONEN); Criola; Geledés Instituto da Mulher Negra; Instituto Cultural Steve Biko; Soweto Organização Negra
Brazil	Indigenous peoples	Catholic Church's Indian Missionary Council (CIMI); Commission for the Creation of the Yanomami Park (CCPY); Coordination of the Indigenous Organisations of the Brazilian Amazon (COIAB); Instituto Socioambiental (ISA); Kanindé (Association for Ethnic-Environmental Defense)
Burundi	Hutu	Conseil national pour la defense de la democratie (CNDD); Conseil national pour la defense de la democratie – Forces pour la defense de la democratie (CNDD–FDD); Forces nationales de liberation (FNL); Front de Libération National (FROLINA) /Umbumwe (Solidarity); Front pour la democratie au Burundi (FRODEBU); Hutu Peoples Liberation Party (PALIPEHUTU) / Burundi Peoples Liberation Party (PALIPE-Agakisa); Ligue Burundaise des Droits de l'Homme (ITEKA); Movement for Peace and Democracy / Mouvement pour la paix et la Démocratie (MPDP / MPD); National Forces of Liberation Icanzo (FNL-Icanzo); Parti du peuple (PP); Rassemblement du peuple du Burundi (RPB); Sahwanya Frodebu-Nyakuri (FRODEBU-Nyakuri)
Burundi	Tutsi	Parti pour le redressement national (PARENA); Ralliement pour la democratie et le developpement economique et sociale (RADDES); Union pour le progres national (UPRONA)
China	Mongolians	Asia-Mongolian Front for Freedom; Bayannur League National Modernization Society; General Coordination Committee of Inner Mongolian Rejuvenation Movements; Ih Ju League National Culture Society; Inner Mongolia National Autonomous Committee; Southern Mongolian Democracy Alliance
China	Tibetans	Government-in-exile in Dharamsala; International Tibet Support Network; Students for a Free Tibet; Tibetan Women's Congress; Tibetan Youth Congress
China	Uyghur	East Turkestan Information Centre; East Turkestan Islamic Party (ETIM); Organization for the Liberation of Uighurstan; True God Party (Zhen Zhu Dang); Uighur Liberation Party; United Association of Uighurs; United National Revolutionary Front of East Turkestan (UNRF); Yizhabute
El Salvador	Indigenous peoples	Asociación Coordinadora de Comunidades Indígenas de El Salvador (ACCIES); Asociación Nacional Indígena Tierra Sagrada (ANITSA); Consejo Coordinador Nacional Indígena Del Salvador (CCNIS); Instituto para el Rescate Ancestral Indigena Salvadoreño (RAIS); Movimiento Autóctono Indígena Salvadoreño (MAIS)
Guinea-Bissau	Balanta	Partido da Renovacao Social (PRS)
Guinea-Bissau	Manjaco	Frente de Luta pela Independencia da Guinee (FLING)
Iraq	Kurds	Ansar al-Islam (AI); Democratic Patriotic Alliance of Kurdistan (DPAK); Freedom and Social Justice List (FSJL); Iraqi Kurdistan Front (IKF) / Kurdistan Front (KF); Islamic Fayli Grouping in Iraq (IFGI); Islamic Group of Kurdistan (IGK); Islamic Union of Kurdistan (IUK); Kurdish Conservative Party (KCP); Kurdish Democratic Party – KDP (al-Hizb al-Dimuqraati al-Kurdi); Kurdish Hezbollah (KH); Kurdish Unity Party (KUP); Kurdistan Communist Party (KCP); Kurdistan Democratic Independence Party (KDIP); Kurdistan Democratic Party (KDP); Kurdistan Islamic

Country	Ethnic group	Organizations
		League (KIL) / Islamic Movement of Iraqi Kurdistan (IMIK); Kurdistan List (KL); Kurdistan National Democratic Union (KNDU); Kurdistan People's Democratic Party (KPDP) / Kurdistan People's Party (KPP); Kurdistan Revolutionary Party (KRP); Kurdistan Socialist Democratic Party (KSDP); Kurdistan Socialist Party (KSP); Kurdistan Toilers Party (KTP); Movement for Change (MC); Party for Independence of Kurdistan (PIK); Patriotic Union of Kurdistan (PUK); Workers' Communist Party of Iraq (WCPI)
Iraq	Shi'a Arabs	Badr Organisation (BO); Hezbollah Movement in Iraq (HMI); Holy Warriors (al-Mujahidin); Imam Soldiers (Jund al-Imam); Iraqi National Alliance (INA); Islamic Action Organization in Iraq (IAOI); Islamic Dawa Party (IDP); Islamic Dawa Party - Iraq Organisation (IDP-IO); Islamic Movement in Iraq (IMI); Islamic Scholars Organization (ISO); Islamic Supreme Council (ISC); Islamic Virtue Party (IVP); National Independent Cadres and Elites (NICE); National Reform Movement (NRM); Progressives (P); Sadrist Movement (SM); State of Law Coalition (SLC); United Iraqi Alliance (UIA)
Iraq	Sunni Arabs	Ansar al-Islam (AI); Arab Socialist Baath Party (ASBP); Iraqi Accord Front (IAF); Iraqi National Accord (INA); Iraqi National Dialogue Front (INDF); Iraqi National Movement (INM); Reconciliation and Liberation Bloc (RLB); Unity Alliance of Iraq (UAI)
Israel	Ashkenazim (Jewish)	Agudat Israel Workers / Poalei Agudat Yisrael [D] (AIW); Association of Israel / Agudat Yisrael [G] (AI); Ta'ayush: Arab-Jewish Partnership; Torah Flag / Degel Hatorah [EZ] (TF); Union [MHL] (Likud); United Torah Judaism / Yahadut Hatorah [G] (UTJ)
Israel	Israeli Arabs	Adalah: The Legal Center for Arab Minority Rights in Israel; Arab Association for Human Rights; Arab Democratic Party (ADP); Arab Movement for Renewal / al-Haraka al-Arabiya lil-Taghyeer / Tnu'a Aravit LeHithadshut (Ta'al); Association for the Defence of Bedouin Rights; Democratic Front for Peace and Equality / Hazit Demokratit Leshalom Veshivayon [W] (Hadash); Ittijah, the network for Palestinian non-govern-mental organizations (NGOs) in Israel; National Democratic Alliance / al-Tajammu al-Watani al-Dimuqrati [D] (Balad); Progressive List for Peace / Hareshima Hamitkademet Leshalom [P] (PLP); Ta'ayush: Arab-Jewish Partnership; United Arab List [AM] (UAL)
Israel	Mizrahim (Jewish)	Bridge / Gesher; Israel Tradition Movement / Tnuah Lemasoret Yisrael [NJ] (Tami); Sephardi Torah Guardians / Shomrei Torah Sfaradim [SHAS] (Shas); Union [MHL] (Likud); Yeminite Association (YA)
Israel	Palestinian Arabs	Abu Nidal Organization / Fatah Revolutionary Council / Black June Organization; Adalah: The Legal Center for Arab Minority Rights in Israel; Al-Aqsa Martyrs' Brigades; Al-Khalas National Islamic Party; Alliance of Palestinian Forces (APF); Arab Association for Human Rights; Arab Liberation Front; Democratic Front for the Liberation of Palestine (DFLP); Fatah / PLO-Lebanon Faction / Fatah Hawks; Fatah Uprising / Omar al-Mokhtar Forces; Hamas / Islamic Resistance Movement; Ibn al-Balad / Sons of the Village; Ittijah, the network for Palestinian non-govern-mental organizations (NGOs) in Israel; Musawa: Palestine Centre for the Independence of the Judiciary and the Legal Profession; New Communist Party / Reshima Kommunistit Hadasha [W] (Rakah); Palestine Liberation Front (PLF) / People's Palestine Liberation Front; Palestine National Salvation Front; Palestinian Centre for Peace and Democracy; Palestinian Democratic Union (FIDA or FEDA); Palestinian Islamic Jihad (PIJ) / Islamic Jihad; Palestinian Liberation Organisation (PLO); Palestinian People's Party; Palestinian Popular Struggle Front (PPSF);

Country	Ethnic group	Organizations
		Popular Front for the Liberation of Palestine (PFLP); Popular Front for the Liberation of Palestine General Command (PFLP-GC); Popular Resistance Committees (PRC) / Sallah el-Dein Brigades (PRC armed wing); Ta'ayush: Arab-Jewish Partnership
Israel	Russians (Jewish)	Immigration Party / Yisrael Baaliya [KN] (IP); Israel Our Home / Yisrael Beitenu [L] (IOH)
Lithuania	Lithuanians	Homeland Union-Lithuanian Christian Democrats (TS-LKD); Liberal and Centre Union (LiCS); Lithuanian Centre Union (LCS); Lithuanian Christian Democratic Party (LKDP); Lithuanian Freedom Union (LLaS); Lithuanian National Party "Young Lithuania" (LNPJL); Lithuanian National Union (LTS); Lithuanian Union of Political Prisoners and Deportees(LPKTS); National Progress Movement (TPJ); Order and Justice (TT); Sajudis Movement
Lithuania	Poles	Alliance of Lithuanian National Minorities (LTMA); Association of Polish Teachers in Lithuania / Macierz Szkolna; Congress of Poles in Lithuania (Kongres Polakow Litwy – KPL); Lithuanian Poles' Electoral Action (AWPL or LLRA); Union of Poles in Lithuania (Zwiazek Polakow na Litwie – ZPL)
Lithuania	Russians	Alliance of Lithuanian National Minorities (LTMA); Union of Russians in Lithuania (LRS)
Macedonia	Albanians	All Albanian Army (AAA); Democratic Party for Albanians (DPA); Democratic Union for Integration (DUI); Ilirida Albanian youth Movement; National Democratic Party (NDP(2)); National Democratic Revival (NDP(3)); National Liberation Army (NLA); New Democracy (ND); New Democratic Forces (FRD); Party for Democratic Prosperity (PDP); Party for Democratic Prosperity of Albanians (PDPA); People's Democratic Party (NDP(1))
Macedonia	Macedonians	People's Movement of Macedonia (NDM); VMRO-DPMNE; World Macedonian Congress
Macedonia	Roma	Party for Full Emancipation of Romas of Macedonia (PCERM); Party for Roma Integration (PIR); Roma Centre of Skopje; Roma Humanitarian Association (Sun) / Roma Democratic Development Association (SONCE); Union of Romas in Macedonia (SRM); United Party for the Emancipation of Roma (OPER)
Macedonia	Serbs	Democratic Party of Serbs in Macedonia (DPSM)
Macedonia	Turks	Democratic Party of Turks in Macedonia (DPTM); Party for the Movement of Turks in Macedonia (PDTM)
Madagascar	Côtiers	AREMA; MFM; MONIMA; PSD
Madagascar	Highlanders	AKFM
Malawi	Central (Chewa)	Malawi Congress Party (MCP); The Movement for the Restauration of Democracy in Malawi (MRDM)
Malawi	Northerners (Tumbuka, Tonga, Ngonde)	Alliance for Democracy (AFORD); Congress of the Second Republic (CSR); Malawi Freedom Movement (MAFREMO); Movement for Genuine Democratic Change (MGODE)
Malawi	Southerners (Lomwe, Mang'anja, Nyanja, Yao)	Democratic Progressive Party (DPP); National Democratic Alliance (NDA); United Democratic Front (UDF)

Country	Ethnic group	Organizations
Malaysia	Chinese	Communist Party of Malaya; Democratic Action Party; Liberal Democratic Party; Malayan Chinese Association; Malaysian People's Movement Party; People's Action Party; Sabah Progressive Party; Sarawak Chinese Association; Sarawak United People's Party
Malaysia	Dayaks	Parti Bumiputera Sarawak; Sarawak Dayak Iban Association; Sarawak National Party; Sarawak Native People's Party
Malaysia	East Indians	Democratic Action Party; Hindu Rights Action Force; Malayan Indian Congress; Malaysian Punjabi Party; Muslim Unity Movement; People's Progressive Party
Malaysia	Kadazans	Kadazandusun Cultural Association; Kadazandusun Language Foundation; Sabah People's United Front; Sabah Progressive Party; United Pasokmomogun Kadazandusun Murut Organisation; United Sabah Party; United Sabah People's Party
Malaysia	Malays	Malaysia Mujahideen Group; Malaysian People's Welfare Party; Malaysian United People's Party; Muslim Unity Movement; National Party; Pan-Malayan Islamic Party; Pan-Malaysian Islamic Front; Semangat 46; United Malays National Organisation
Mozambique	Makonde-Yao	Frente de Libertacao de Mocambique (FRELIMO)
Mozambique	Shona-Ndau	Resistencia Nacional Mocambicana (RENAMO)
Mozambique	Tsonga-Chopi	Frente de Libertacao de Mocambique (FRELIMO)
Myanmar	Bamar (Barman)	UDLDP – Union Danu League for Democracy Party
Myanmar	Buddhist Arakanese	AA – Arakan Army; AIO – Arakan Independence Organisation; ALD – Arakan League for Democracy / RDL – Rakhine Democracy League; ALP – Arakan Liberation Party; ANC – Arakan National Council; ANUO – Arakan National Unity Organisation / IAPG – Independent Arakanese Parliamentary Group; APDF – Arakan People's Democratic Front; DAB – Democratic Alliance of Burma; NDF – National Democratic Front; NUPA – National United Party of Arakan; RNPP – Rakhine National Progressive Party; TNA – Tribal National Party; UNLD – United Nationalities' League for Democracy
Myanmar	Kachins	AKSYU – All Kachin Student and Youth Union; DAB – Democratic Alliance of Burma; KDA – Kachin Defense Army; KIO – Kachin Independence Organization / KIA – Kachin Independence Army; KNC – Kachin National Congress; KPP – Kachin People's Party; KSNCD – Kachin State National Congress for Democracy; KSNLD – Kachin State Nationalities League for Democracy; Kachin Development Networking Group; Kachin Women’s Association – Thailand; NDF – National Democratic Front; UDPKS – Unity and Democracy Party of Kachin State; UNLD – United Nationalities' League for Democracy
Myanmar	Karenni (Red Karens)	DAB – Democratic Alliance of Burma; DOKNU – Democratic Organisation for Kayah National Unity; KDL – Kayah Democratic League; KNPLF – Karenni Nationalities People's Liberation Front; KNPP – Karenni National Progressive Party; KNUL – Kayah National Unity League; KRA – Karenni Revolutionary Army; Karenni Independence through Education; NDF – National Democratic Front; UNLD – United Nationalities' League for Democracy
Myanmar	Kayin (Karens)	Committee for Internally Displaced Karen People; DAB – Democratic Alliance of Burma; DKNO – Democratic Karen Buddhist Organization / DKBA – Democratic Karen Burmese Army; Friends of the Karen: People of Burma; God's Army; KNPP – Karen National Progressive Party; KNU – Karen National Union; KNUP – Karen National United Party; KPP – Kayin People's Party; KSNO – Karen State National

Country	Ethnic group	Organizations
		Organisation; Karen Human Rights Group; NDF – National Democratic Front; SSNLO – Shan State Nationalities Liberation Organisation; UNLD – United Nationalities' League for Democracy
Myanmar	Mons	AMRDP – All Mon Region Democracy Party; DAB – Democratic Alliance of Burma; Human Rights Foundation of Monland; MNDF – Mon National Democratic Front; MNF – Mon National Front; Monland Restoration Council; NDF – National Democratic Front; NMSP – New Mon State Party / MNLA – Mon National Liberation Army; UNLD – United Nationalities' League for Democracy
Myanmar	Muslim Arakanese	AA – Arakan Army; ANC – Arakan National Council; ARIF – Arakan Rohingya Islamic Front; ARNO – Arakan Rohingya National Organisation; KNLD – Kaman National League for Democracy; Kawthoolei Muslim Patriotic Front; NDPHR – National Democratic Party for Human Rights; NUPA – National United Party of Arakan; RNA – Rohingya National Army; RSO – Rohingya Solidarity Organization; Rohingya Pariotic Front
Myanmar	Shan	ASSO – All–Shan State Organization; DAB – Democratic Alliance of Burma; Mong Tai Army; NDF – National Democratic Front; SDU – Shan Democratic Union; SNDP – Shan National Democratic Party; SNLD – Shan Nationalities League for Democracy; SNUF – Shan National United Front; SSA – Shan State Army (Old Faction) / SSPP – Shan State Progressive Party; SSA–MTA – Shan State Army – Mong Tai Army faction / SUA – Shan United Army / SSNC – Shan State National Congress; SSA–S – Shan State Army–South / SURA – Shan United Revolutionary Army / SSRC– Shan State Restoration Council; SSIA – Shan State Independence Army; SSKDP – Shan State Kokang Democratic Party; SSNA – Shan State National Army; SSPO – Shan State Peasants' Organisation / SSUHPO – Shan State United Hill People's Organisation; Shan Human Rights Foundation; Shan Relief and Development Committee; Shan State Revolutionary Army; Shan Women's Action Network; UHPC – United Hill People's Congress; UNLD – United Nationalities' League for Democracy
Myanmar	Wa	DAB – Democratic Alliance of Burma; NDF – National Democratic Front; UNLD – United Nationalities' League for Democracy; UWSA – United Wa State Army / MNSP – Myanmar National Solidarity Party; WDP – Wa Democratic Party; WNA – Wa National Army; WNO – Wa National Organization
Myanmar	Zomis (Chins)	CHRO - Chin Human Rights Organization; CNF – Chin National Front / CNA – Chin National Army; CNLD – Chin National League for Democracy; CNO – Chin National Organisation / CNP – Chin National Party; CPP – Chin Progressive Party; Chin Forum; DAB – Democratic Alliance of Burma; MKNSO – Mro (Khami) National Solidarity Organisation; NDF – National Democratic Front; UNLD – United Nationalities' League for Democracy; Women's League of Chinland; ZLF – Zomi Liberation Front; ZNC – Zomi National Congress; Zomi Re-unification Organization
Turkey	Kurds	Baris ve Demokrasi Partisi (Peace and Democracy Party); Demokratik Halk Partisi (Democratic People's Party); Demokratik Toplum Partisi (Democratic Society Party); Halkin Demokrasi Partisi (People's Democracy Party); Halkin Emek Partisi (People's Labor Party) (HEP) - Demokrasi Partisi (Democracy Party) (DEP); Kurdish Parliament in Exile in the Hague; Kurdish Workers Party (PKK-KADEK); Kurdistan Liberation Hawks (TAK); Kurdistan National Congress (KNK); Kurdistan Worker's Party - Revolutionary Line Fighters / Partiya Karkeren Kurdistan - Cevrimci Cizgi Savascilari (PKK-DCS); Party of God / Turkish Hizbullah /

Country	Ethnic group	Organizations
		Hizbullah-Contras / Kurdish Hizbullah / Hür Dava Partisi (Huda-Par) (Free cause party); Rights and Freedoms Party (HAK-PAR)
Turkey	Turkish	Grey Wolves / National Task Force; Milliyetci Hareket Partisi (Nationalist Action Party); Turkish Avenger Brigade / Türk Intikam Tugayi (TIT)
Pakistan	Baluchis	Balochistan National Movement (BNM); Balochistan National Party (BNP); Baluch Ittehad; Baluch Liberation Army; Baluch People's Liberation Front; Baluch Students Organization (BSO); Baluchistan National Alliance (BNA); Jamhoori Wattan Party/Democratic Motherland Party (JWP); Pakistan Oppressed Nations Movement (PONM); Sindh-Baluchistan Patriotic Front
Pakistan	Bengali	Awami League/People's League (AL); Bangladeshi Consultative Committee; Communist Party of Bangladesh; Mukhti Bahini (Liberation Army); Provisional Government of Bangladesh
Pakistan	Christians	Christian Liberation Front (CLB)
Pakistan	Mohajirs	Muttahida Qaumi Movement/United National Movement (MQM)
Pakistan	Pashtuns	Awami National Party/People's National Party (ANP); Pakistan Oppressed Nations Movement (PONM); Pakistan Tehreek-e-Insaaf/Pakistan Justice Movement (PTI); Pukhtoonkhwa Milli Awami Party/Pakhtoon National People's Party (PKMAP); Sindh-Baluchistan Patriotic Front
Pakistan	Sindhi	Jaye Sindh Progressive Party; Jaye Sindh Student Federation (JSSF); Jeay Sindh Qaumi Mahaz (JSQM); Jiye-Sindh Qaum Parast Part (JSQPP); Pakistan Oppressed Nations Movement (PONM); Pakistan People's Party (PPP); Sind National Front (SNF); Sindh National Alliance; Sindh National Party (SNP); Sindh Taraqi Pasand Party; Sindh-Baluchistan Patriotic Front
Paraguay	Tupi-Guaraní and other indigenous groups	Asociación de Parcialidades Indígenas (API); Gente, Ambiente y Territorio (GAT); Tierra Viva
Peru	Afroperuvians	Asociación Negra de Defensa y Promoción de los Derechos Humanos (ASONEDH); Centro de Desarrollo de la Mujer Negra Peruana (CEDEMUNEP)
Peru	Indigenous peoples of the Amazon	Asociación para la Conservación del Patrimonio del Cutivireni (ACPC); Centro de Investigación y Promoción Amazónica (CIPA); Comisión Andina de Juristas (CAJ) / Andean Commission of Jurists; Confederation of Amazonian Nationalities of Peru (CONAP); Inter-Ethnic Association for the Development of the Peruvian Jungle (AIDESEP); La Confederación de la Nacionalidades Indígenas de Perú (CONAIP); Partido Nacionalista Peruano (PNP) / Peru Nationalist Party; Regional Indigenous Organization of Atalaya (OIRA)
Peru	Indigenous peoples of the Andes	Comisión Andina de Juristas (CAJ) / Andean Commission of Jurists; La Confederación de la Nacionalidades Indígenas de Perú (CONAIP); Partido Nacionalista Peruano (PNP) / Peru Nationalist Party
Russia	Abkhaz	Caucasian confederation / Confederation of the Caucasian (Mountain) peoples (KGNK)
Russia	Adyghe	Caucasian confederation / Confederation of the Caucasian (Mountain) peoples (KGNK)
Russia	Avars	Caucasian confederation / Confederation of the Caucasian (Mountain) peoples (KGNK); Nur (Light); Union of Muslims of Russia (Soyuz Musulman Rossi)

Country	Ethnic group	Organizations
Russia	Buryats	Buryat-Mongolian National Party / Buryat-Mongolian Peoples' Party; Erkhe; Regional Union of Young Scholars
Russia	Byelorussians	The Federal National-Cultural Autonomy of the Belarusians of Russia
Russia	Chechens	(All-) National Congress of Chechen People ((A)NCCP, OKChN) / Dudayev Govt / Chechen National Congress / Dudayev's forces; Assembly for the Defense of the Sovereignty (of the CRI); Caucasian confederation / Confederation of the Caucasian (Mountain) peoples (KGNK); Daymokhk (Fatherland / "our land" / DAIMOHK); Gen Dzhokhar Dudayev's army / Salman Raduyev's rebels; Islamic Special Purpose Regiment / Islamic Regiment; Kadyrovs (Kadyrovtsy); Maskhadov's rebel group; Noy Worldwide Chechen Fund; Nur (Light); Ruslan Labazanov's rebels; Shamil Basayev rebels; Union of Citizens for the Chechen Republic as a Democratic Rule-of-Law State Wit (Solidarity); Union of Patriotic Forces (UOP)
Russia	Cherkess	Caucasian confederation / Confederation of the Caucasian (Mountain) peoples (KGNK)
Russia	Chukchi	Russian Association of Indigenous Peoples of the North
Russia	Dargins	Caucasian confederation / Confederation of the Caucasian (Mountain) peoples (KGNK)
Russia	Jews	Federation of Russian Jewish Communities of the CIS
Russia	Kabardins	Caucasian confederation / Confederation of the Caucasian (Mountain) peoples (KGNK)
Russia	Kumyks	Tenglik Movement
Russia	Laks	Caucasian confederation / Confederation of the Caucasian (Mountain) peoples (KGNK); Union of Muslims of Russia (Soyuz Musulman Rossi)
Russia	Lezgins	Lezgin National Council (National Council of the Lezghin People); Sadval / Lezgin National Movement (including Sadval extremist wing and Sadval moderate wing); Union of Muslims of Russia (Soyuz Musulman Rossi)
Russia	Ossetes	Caucasian confederation / Confederation of the Caucasian (Mountain) peoples (KGNK)
Russia	Russians	Pamyat (Memory); Rebirth (Vozrozhdeniye); Zhirinovsky bloc / Political Party LDPR / Liberal Democratic Party of Russia
Russia	Tatars	All-Tatar Public Centre (VTOTs) / Tatar Public Center (TOTs, TPC) / All-Tatar Social Centre (ATSS); Azatlyk Union of the Tatar Youth (Azatlyk (Freedom)); Ittifak Party / Tatar Independence Party / Ittifaq; Muslims of Russia (Musulmani Rossi); Nur (Light); Suverenitet (Sovereignty Committee); Tatar National Congress (Milli Majlis/Mejlis); Tatar National Front (TNF); Union of Muslims of Russia (Soyuz Musulman Rossi)
Russia	Tuvinians	Russian Association of Indigenous Peoples of the North
Serbia	Albanians	Democratic Union of the Valley (BDL); Movement of Democratic Progress (PDP); Party of Democratic Action (PDD)
Serbia	Bosniak/Muslims	Bosniak National Council (BNVS); Coalition "All together"; List for Sandzak (LzS); Party for democratic action of Sandzak (SDAS); Sandzak Democratic Party (SDP)
Serbia	Croats	Coalition "All together"

Country	Ethnic group	Organizations
Serbia	Hungarians	Coalition “All together”; Democratic Community of Hungarians in Vojvodina (DZVM); Hungarian Democratic Party of Vojvodina (DSVM); Union of Vojvodina Hungarians (SVM)
Serbia	Roma	Roma Party (RP); Roma Union of Serbia (URS1); Roma Women's Center BIBIJA
Serbia	Serbs	Serb Assembly Dveri; Serbian Radical Party (SRS)
South Sudan	Dinka	Sudan People's Liberation Movement/Army (SPLM/A)
South Sudan	Nuer	Sudan People's Liberation Movement/Army (SPLM/A); United Democratic Front (UDF)
South Sudan	Shilluk	Sudan People's Liberation Movement - Democratic Change (SPLM-DC)
Namibia	Baster	Federal Convention of Namibia (FCN); United People's Movement (UPM)
Namibia	Damara	United Democratic Front (UDF)
Namibia	Herero, Mbanderu	National Unity Democratic Organization (NUDO)
Namibia	Kavango	All People's Party (APP)
Namibia	Mafwe	Caprivi Liberation Army (CLA); Caprivi Liberation Front (CLF); United Democratic Party (UDP)
Namibia	Ovambo	South West Africa People's Organization (SWAPO)
Namibia	San	Nyae Nyae Development Foundation of Namibia (NNDFN)
Namibia	Whites	Aksie Christelik Nasionaal (ACN); Monitor Action Group (MAG); Republican Party (RP)
Spain	Basques	Amaiur; Batasuna; Behatokia; Comandos Autonomos Anticapitalistas (CCA); ETXERAT; Euzkadi ta Azkatasuna (ETA); Herri Batasuna (HB); Partido Nacionalista Vasco (EAJ-PNV)
Spain	Catalans	Academia de Cultura Valenciana; Acció Cultural Pais Valencia; Convergencia i Unio (CiU); Esquerra Republicana de Catalunya (ERC); Institut d’Estudis Catalans; Moviment de Defensa de la Terra (MDT); Pacte Democratic per Catalunya (PDPC); Terra Lliure (TL)
Spain	Galician	A Mesa pola Normalización Lingüística; Bloque Nacionalista Galego (BNG); Exército Guerrilheiro do Povo Galego Ceive (EGPGC)
Spain	Roma	Asociación de Enseñantes con Gitanos; Fundación Secretariado Gitano; Presencia Gitana; Unión Romaní
Sri Lanka	Indian Tamils	All Ceylon Tamil Congress (ACTC); Ceylon Workers' Congress (CWC); Democratic People's Liberation Front (DPLF); Eelam People's Revolutionary Liberation Front (EPRLF); Eelam Revolutionary Organization of Students (EROS); Eelavar Democratic Front (EDF); Federal Party (Illankai Thamil Arasu Kadchi, FP/ITAK); National Union of Workers (NUW); Tamil United Liberation Front (TULF/TUF); Up-Country People's Front (UPF)
Sri Lanka	Moors (Muslims)	All Ceylon Muslim Congress (ACMC); All Ceylon Tamil Congress (ACTC); Eelam Revolutionary Organization of Students (EROS); Eelavar Democratic Front (EDF); Muslim National Unity Alliance (MNUA); National Congress (NC); Sri Lanka Muslim Congress (SLMC)

Country	Ethnic group	Organizations
Sri Lanka	Sinhalese	Democratic United National Front (DUNF); Jathika Hela Urumaya (JHU, National Heritage Party); Jathika Nidahas Peramuna (JNP, National Freedom Front); Jathika Vimukti Peramuna (JVP, People's Liberation Front); Sinhalese Heritage; Sons of the Soil Party; Sri Lanka Freedom Party (SLFP); Sri Lanka Freedom Socialist Party (SLFSP); United National Party (UNP)
Sri Lanka	Sri Lankan Tamils	All Ceylon Tamil Congress (ACTC); Democratic People's Front (DPF); Democratic People's Liberation Front (DPLF); Eelam National Liberation Front (ENLF); Eelam People's Democratic Party (EPDP); Eelam People's Revolutionary Liberation Front (EPRLF); Eelam Revolutionary Organization of Students (EROS); Eelavar Democratic Front (EDF); Federal Party (Illankai Thamil Arasu Kadchi, FP/ITAK); Liberation Tigers of Tamil Eelam (LTTE); People's Liberation Organization of Tamil Eelam (PLOTE); People’s Front of the Liberation Tigers (PFLT); Tamil Eelam Army; Tamil Eelam Liberation Army (TELA); Tamil Eelam Liberation Organisation (TELO); Tamil Makkal Viduthalai Pulikal (TMVP); Tamil United Liberation Front (TULF/TUF)
Taiwan	Indigenous/Aboriginal Taiwanese	Democratic Progressive Party; Taiwan Aboriginal Tribes Association (TATS)/Alliance of Taiwan Aborigines
Taiwan	Mainland Chinese	Chinese Youth Party (CYP); Kuomintang (Nationalist Party)
Taiwan	Taiwanese	Democratic Progressive Party; Formosa Party; Taiwan Independence Party (TAIP)
Tanzania	Maasai	Community Research and Development (CORD); KIPOC / KIPOC-Barabaig
Tanzania	Mainland Africans	Afro-Shirazi Party (ASP); Democratic Party (DP)
Tanzania	Mainland Muslims	Civic United Front (CUF); Council of the Dissemination of the Quran in Tanzania / Balukta
Tanzania	Others Mainland (Christians and traditional religions)	Democratic Party (DP)
Tanzania	Shirazi (Zanzibar Africans)	Civic United Front (CUF); Zanzibar and Pemba People's Party (ZPPP)
Tanzania	Zanzibar Arabs	Bismallah (“In the name of God”) / Bismillahi Movement; Civic United Front (CUF); Zanzibar Nationalist Party (ZNP); Zanzibar Organization
Trinidad and Tobago	Blacks	African National Congress (ANC); Democratic Action Congress (DAC); People's National Movement (PNM); Tapia House Movement (THM)
Trinidad and Tobago	East Indians	Democratic Labour Party (DLP); United Labour Front (ULF); United National Congress (UNC)
Yemen	Northerners	People's General Congress (PGC)
Yemen	Southerners	Free Yemen Party (FYP); League of the Sons of Yemen (LSY); Southern Movement (Hirak); Yemeni Socialist Party (YSP)
Yemen	Sunni Shafi'i (Arab)	Aden-Abyan Islamic Army (AAIA); Al-Rashad Union Party; Yemeni Congregation for Reform (al-Islah)
Yemen	Zaydis	Nation Party (Hizb al-Omma); Party of Truth (al-Haqq)
Zimbabwe	Africans	National People's Party (NPP); Patriotic Front - Zimbabwe African People's Union / Zimbabwe People's Revolutionary Army (PF-ZAPU/ZIPRA); Zimbabwe African

Country	Ethnic group	Organizations
		National Union - Patriotic Front / Zimbabwe African National Liberation Army (ZANU-PF/ZANLA)
Zimbabwe	Manyika (Shona sub-group)	Zimbabwe African National Union - Patriotic Front / Zimbabwe African National Liberation Army (ZANU-PF/ZANLA)
Zimbabwe	Ndau (Shona sub-group)	Zimbabwe African National Union - Ndonga (ZANU-Ndonga); Zimbabwe African National Union - Patriotic Front / Zimbabwe African National Liberation Army (ZANU-PF/ZANLA)
Zimbabwe	Ndebele-Kalanga-(Tonga)	Movement for Democratic Change - Mutambara/Ncube (MDC-M/N); Patriotic Front - Zimbabwe African People's Union / Zimbabwe People's Revolutionary Army (PF-ZAPU/ZIPRA); United National Federal Party (UNFP); United People's Association (of Matabeleland); ZAPU2000
Zimbabwe	Shona	Chimwenje; Zimbabwe African National Union - Ndonga (ZANU-Ndonga); Zimbabwe African National Union - Patriotic Front / Zimbabwe African National Liberation Army (ZANU-PF/ZANLA)
Zimbabwe	White Zimbabweans	Independent Zimbabwe Group (IZG); Republican Alliance (RA); Rhodesian Front / Republican Front / Conservative Alliance of Zimbabwe (RF/RF/CAZ)

Notes: The list of ethnic groups follows EPR 2014 and is time-variant (e.g., groups may split over time). This means that the groups listed in the table for each country are not always politically relevant at the same time.

Appendix II: EPR-Organizations (EPR-O) and Its Alternatives

	**EPR-O**	**MAROB**	**SRDP**
Geographic coverage	Global (random sample)	Middle East and North Africa	Global (countries with self-determination movements)
Temporal coverage	1946-2013	1980-2004	1960-2005
N countries	40	16	77
N organizations	668	118	1,124
Avg. org/country	16.7	7.4	14.6
Coding of organizational demands	Yes (6 different types of demands)	Yes (3 motivation and 11 ideology variables)	Yes (5 different types of territorial demands)

Notes: MAROB = Minorities at Risk Organizational Behavior; SRDP = Strategies of Resistance Data Project. "Coding of organizational demands" for SRDP according to original SDM dataset (Cunningham 2013).

Appendix III: Descriptive Statistics

Table A1: Summary statistics of the independent variables, group-level

Variable	N	Mean	Median	Std. Dev.	Min	Max
Scope of demands, maximum organizational value	12,613	.34	0	.73	0	3
Scope of demands, median organizational value	12,613	.23	0	.55	0	3
N organizations	12,613	1.35	0	2.56	0	25
Relative group size	12,613	.15	.02	.25	0	.97
N of TEK connections	12,613	3.10	1	5.08	0	27
Geographic concentration	12,613	.81	1	.40	0	1
Excluded	12,613	.74	1	.44	0	1
Regional autonomy	12,613	.44	0	.50	0	1
Downgraded in last 5 years	12,613	.04	0	.19	0	1
Liberal democracy	12,361	.20	.09	.22	.02	.87
GDP per capita (logged)	12,534	7.96	8.14	1.26	4.63	10.67
N of years of mobilization	12,613	9.61	0	15.63	0	68
Use of small-scale violence by movement	12,613	.09	0	.29	0	1
Group’s conflict history	12,613	.17	0	.58	0	5
N of other groups in country	12,613	24.35	11	23.19	1	57
Country population (logged)	12,534	17.74	18.01	2.09	13.20	21.02
N of religious segments	12,613	1.79	2	.78	0	3
N of linguistic segments	12,613	1.42	1	.71	0	3

Table A2: Summary statistics of the independent variables, organization-level

Variable	N	Mean	Median	Std. Dev.	Min	Max
Radicalization of organization	14,249	.01	0	.10	0	1
N other organizations in the same movement	14,833	4.68	4	4.31	0	20
Organization age	14,833	18.67	14	17.48	0	119
Use of small-scale violence by organization	14,833	.17	0	.37	0	1

Figure A1: Demands by ethno-political organizations over time

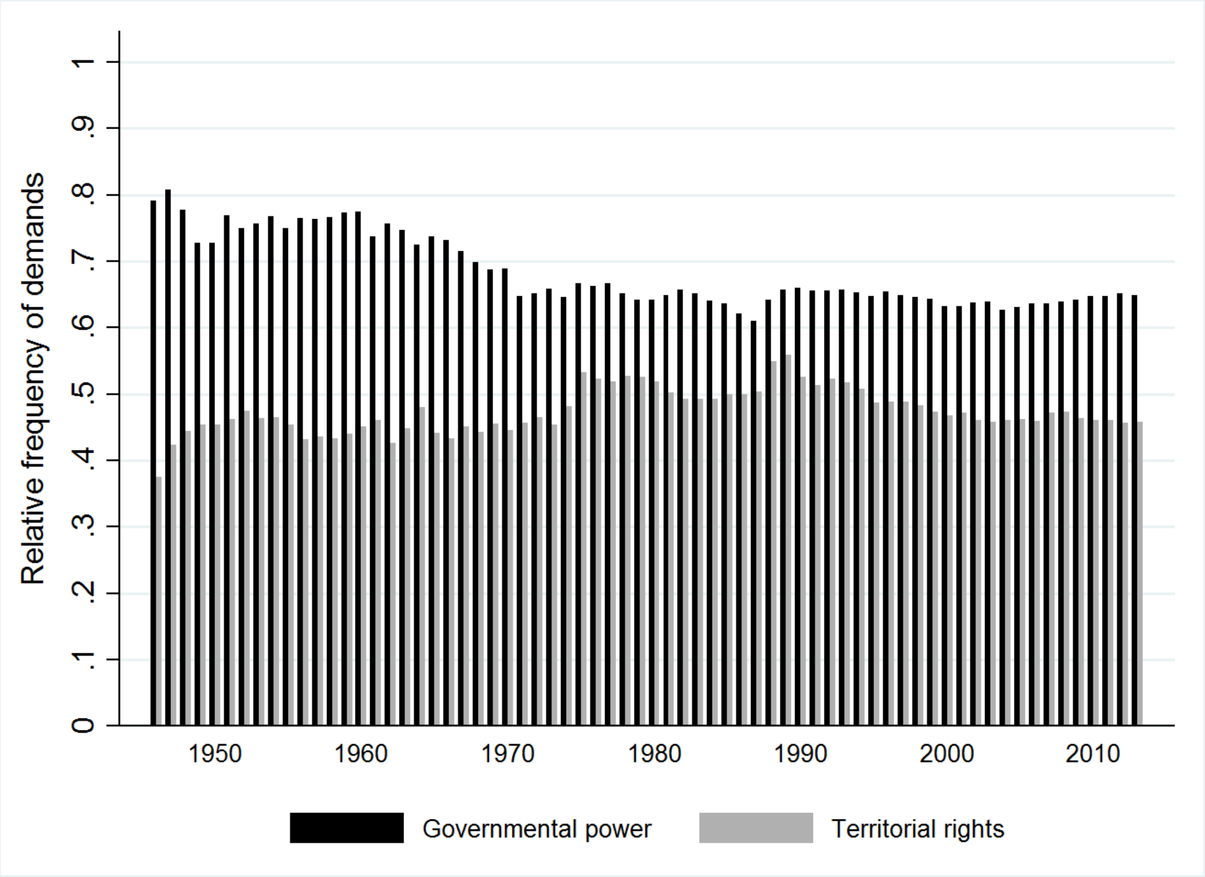

Notes: Unit of analysis is the organization year.

Appendix IV: Movement Fragmentation and the Scope of Demands

Table A3 addresses concerns of endogeneity in the postulated relationship between movement fragmentation and the scope of claims. Model A1 introduces ethnic group-level fixed effects to neutralize unobserved heterogeneity at the movement level. We use the BUC-τ estimator developed by Baetschmann et al. (2020). (Note that the implementation of the estimator "blows up" the sample size by including multiple copies of each observation to perform the necessary dichotomizations of the ordered dependent variable at different cutoff points (Baetschmann et al. 2020, 256-8). Group-level variables with no time variance are omitted from the model.) The effect of the fragmentation variable remains robust.

Next, we address concerns of reverse causality in the relationship between movement fragmentation and the scope of claims, using dynamic versions of the two variables in rare-events logistic regression models. These are dummy variables that are coded as 1 if the number of organizations and the scope of claims, respectively, *increased* within the three foregoing years (i.e. from t_{-4} to t_{-3} or from t_{-3} to t_{-2} or from t_{-2} to t_{-1}). Models A2 and A3 test the effect of an increase in the number of organizations on the likelihood of an increase in the scope of demands in a given year (t). Conversely, Models A4 and A5 evaluate the impact of an increase in the scope of demands in the three preceding years on the likelihood of an increase in the number of organizations. Models A2 and A4 comprise all ethnic group years, whereas Models A3 and A5 restrict the sample to ethnic group years with at least one political organization recorded in our dataset. In the case of reverse causality, we would expect a positive effect of the dynamic radicalization variable in Models A4 and A5. However, the results do not sustain this assumption. By contrast, Models A2 and A3 provide further evidence for our argument that fragmentation

increases the scope of movement demands as a previous increase in the number of organizations has a significant positive impact on the likelihood of radicalization.

Table A3: Movement Fragmentation and the Scope of Demands. Robustness Tests I

	**Model A1**<br>Scope of Demands	**Model A2**<br>Radicalization	**Model A3**<br>Radicalization	**Model A4**<br>Increase in fragmentation	**Model A5**<br>Increase in fragmentation
N organizations (logged)	3.62***<br>(1.08)				
Increase in fragmentation (in three preceding years)		.78**<br>(.29)	.77**<br>(.29)		
Radicalization (in three preceding years)				.27<br>(.21)	.16<br>(.19)
Relative group size		1.04<br>(.54)	.93<br>(.94)	.17<br>(.26)	.12<br>(.29)
N of TEK connections (logged)		.06<br>(.14)	.11<br>(.19)	.09<br>(.07)	.10<br>(.06)
Geographic concentration		.48<br>(.31)	-.01<br>(.39)	.38*<br>(.15)	.08<br>(.17)
Excluded	3.89***<br>(.88)	1.36***<br>(.38)	1.60**<br>(.51)	.18<br>(.15)	.23<br>(.14)
Regional autonomy	-4.31***<br>(.87)	-.78<br>(.42)	-1.59***<br>(.44)	-.07<br>(.19)	-.09<br>(.17)
Downgraded in last 5 years	-.29<br>(.33)	1.84***<br>(.17)	2.38***<br>(.25)	-.31<br>(.25)	-.25<br>(.25)
Liberal democracy	2.95<br>(1.90)	.47<br>(.67)	-1.43<br>(.87)	.84*<br>(.33)	.27<br>(.29)
GDP per capita (logged)	1.01*<br>(.48)	-.14<br>(.11)	.00<br>(.14)	-.03<br>(.07)	.04<br>(.05)
N of years of mobilization	-.05<br>(.03)	.01<br>(.01)	.01<br>(.01)	.01<br>(.01)	.01**<br>(.01)
Use of small-scale violence by movement	.82<br>(.67)	-.91*<br>(.36)	-.78<br>(.47)	.71***<br>(.17)	.62***<br>(.17)
Group's conflict history	.14<br>(.31)	.49**<br>(.16)	.24<br>(.15)	.11<br>(.08)	.05<br>(.05)
N of other groups in country (logged)		-.17<br>(.15)	.22<br>(.23)	-.25***<br>(.07)	-.11<br>(.08)
Scope of demands of other groups	.53<br>(.74)	.43<br>(.25)	.12<br>(.29)	.21*<br>(.09)	-.04<br>(.08)
Country population (logged)	-.77<br>(.74)	-.02<br>(.13)	.07<br>(.17)	-.02<br>(.06)	.01<br>(.06)
Calendar year		-.00<br>(.01)	-.01<br>(.01)	.00<br>(.00)	-.01**<br>(.00)
N of religious segments		.19<br>(.13)	.20<br>(.23)	-.11<br>(.08)	-.24**<br>(.09)
N of linguistic segments		-.15	-.38	-.04	-.06

		(.18)	(.26)	(.10)	(.08)
Cubic polynomial of outcome variable	-	Yes	Yes	Yes	Yes
Constant		-.95<br>(17.21)	16.50<br>(20.93)	-8.50<br>(8.71)	23.58**<br>(7.60)
Cut 2	3.52***<br>(.42)				
Cut 3	7.12***<br>(.77)				
Ethnic-group fixed effects	Yes	No	No	No	No
N	46,350	11,993	4,754	12,109	5,004
Log likelihood	-9350.37***	-494.01***	-275.08***	-1757.35***	-1314.40***

Note: Standard errors in parentheses. Clustering on countries in Models A2-A5. Log-likelihood figures in Models A2-A5 obtained from standard logistic regressions. * $p < 0.05$, ** $p < 0.01$, *** $p < 0.001$.

Models A6-A8 in Table A4 replicate Models 1-3 of Table 2 in the main text, using the median, rather than the maximum, organizational scope value for the outcome variable. The effect of the fragmentation variable remains positive and statistically significant. Thus, internal fragmentation seems to shift ethnic movements as a whole towards more radical claims, rather than simply producing radical outliers.

Table A4: Movement Fragmentation and the Scope of Demands. Robustness Tests II

	Model A6	Model A7	Model A8
N organizations (logged)	.11***<br>(.03)	.14***<br>(.04)	.14**<br>(.05)
Relative group size	-.58*<br>(.28)	.20<br>(.47)	-.40<br>(.57)
N of TEK connections (logged)	.07<br>(.08)	-.08<br>(.12)	-.09<br>(.15)
Geographic concentration	.31<br>(.31)	.77*<br>(.36)	.95**<br>(.32)
Excluded		1.29***<br>(.27)	1.45***<br>(.31)
Regional autonomy		-1.03***<br>(.31)	-1.79***<br>(.39)
Downgraded in last 5 years		1.13***<br>(.21)	1.65***<br>(.26)
Liberal democracy		.45	-.40

		(.47)	(.46)
GDP per capita (logged)		-.01	.10
		(.09)	(.08)
N of years of mobilization		.01	.00
		(.01)	(.01)
Use of small-scale violence by movement		-.46*	-.33
		(.20)	(.25)
Group’s conflict history		.17	.11
		(.09)	(.09)
N of other groups in country (logged)		-.04	.22
		(.11)	(.14)
Scope of demands of other groups		.25	.01
		(.14)	(.19)
Country population (logged)		.01	.14
		(.10)	(.09)
Calendar year		-.01	-.01*
		(.01)	(.01)
N of religious segments		.07	.03
		(.11)	(.15)
N of linguistic segments		-.12	-.15
		(.11)	(.14)
k-1 lagged outcome dummy variables	Yes	Yes	Yes
Cut 1	5.32***	-4.00	-16.00
	(.46)	(14.58)	(11.24)
Cut 2	12.06***	3.15	-7.80
	(.55)	(14.40)	(11.03)
Cut 3	18.89***	10.42	.19
	(.70)	(14.58)	(11.16)
N	12,403	12,299	5,060
Log likelihood	-1056.28***	-995.27***	-654.03***

*Note: Robust standard errors, with clustering on countries, in parentheses. * $p < 0.05$, ** $p < 0.01$, *** $p < 0.001$.*

Appendix V: Movement Demands and Ethnic Civil Conflict Onset

Table A5 provides examples for the two different ways in which the scope of demands affects the risk of violent escalation in the dyadic government-movement interaction. Some ethnic movements advance their maximum demands at the very beginning of the mobilization process whereas others gradually increase their demands over the course of mobilization. Focusing on first-time conflict outbreaks (thus ignoring cases of conflict recurrence), the table lists the ethnic group involved in the conflict, the year of conflict outbreak, the scope of the movement's demands both at the outset of mobilization and in the year before conflict outbreak, as well as the number of years of mobilization before conflict erupted.

Table A5: Movement demands and ethnic civil conflict: examples

Country	Ethnic group	Year	Scope of demands		Years of
			Before outbreak	1st year of mobilization	mobilization
Conflicts without increase in movement demands					
Spain	Basques	1978	3	3	33
Israel	Palestinian Arabs	1965	3	3	9
Myanmar	Kayin (Karens)	1949	2	2	2
Angola	Cabindan Mayombe	1991	2	2	17
Pakistan	Baluchis	1974	2	2	11
Macedonia	Albanians	2001	1	1	11
Bosnia and Herzegovina	Croats	1993	1	1	2
Burundi	Hutu	1965	1	1	4
Bangladesh	Tribal-Buddhists	1975	1	1	4
Zimbabwe	Africans	1967	1	1	3
China	Uyghur	2008	1	1	13
Angola	Bakongo	1991	1	1	17
Conflicts with increase in movement demands					
Sri Lanka	Sri Lankan Tamils	1984	3	2	37
Turkey	Kurds	1984	3	2	7
Pakistan	Bengali	1971	3	2	24
Pakistan	Mohajirs	1990	2	1	7
Russia	Chechens	1994	2	1	49
Iraq	Shi'a Arabs	1982	1	0	25
Yemen	Southerners	1994	1	0	4
Trinidad & Tobago	Blacks	1990	1	0	29

Table A6 provides additional robustness tests for the relationship between movement demands and ethnic civil conflict onset. First, we show that the effect of the scope of demands pertains to both the governmental and territorial-rights dimensions (Model A9). We find the same result when using dummy variables for each unit move in our two-dimensional distance matrix instead of the ordinal indicators (Model A10). Second, the results remain robust when we restrict the sample to ethnic group years with at least one formal political organization recorded in our dataset (Model A11). Third, we test our scope indicator against dummy variables that record whether an ethnic movement made demands for separatism or advanced religious claims, according to EPR-O (Model A12). While neither of these two variables exerts a significant effect on the outcome variable, our results remain robust. Finally, Model A13 relies on the first-year scope indicator, which should mitigate potential concerns of reverse causality. The results confirm the direct effect of the scope of movement demands on ethnic civil conflict outbreak: the larger the distance between the demands and the status quo in the first year of group mobilization, the higher the risk of subsequent conflict outbreak.

Table A6: Movement Demands and Ethnic Civil Conflict Onset. Robustness Tests I

	**Model A9**	**Model A10**	**Model A11**	**Model A12**	**Model A13**
Scope of demands, governmental power	1.33***<br>(.38)				
Scope of demands, territorial rights	.53*<br>(.23)				
1-unit distance dummy, governmental power		1.25***<br>(.38)			
1-unit distance dummy, territorial rights		.93^{+}<br>(.52)			
2-units distance dummy, territorial rights		1.05*<br>(.48)			
Scope of demands			.55**<br>(.18)	.58*<br>(.24)	
1st-year scope of demands					.68***<br>(.12)

N organizations (logged)	.26*	.23*	.30**	.33**	.38***
	(.11)	(.11)	(.12)	(.10)	(.09)
Relative group size	.22	.29	.02	.20	-.10
	(.75)	(.77)	(.97)	(.80)	(.88)
N of TEK connections	.32*	.28^{+}	.08	.29*	.46**
	(.14)	(.15)	(.14)	(.12)	(.17)
Geographic concentration	.80	.71	.15	.66	.76
	(.65)	(.66)	(.63)	(.65)	(.71)
Separatist claim				.48	
				(.39)	
Religious claim				-.03	
				(.32)	
Cubic polynomial of peace years	Yes	Yes	Yes	Yes	Yes
Constant	-4.51***	-4.46***	-3.30***	-4.29***	-4.60***
	(.58)	(.57)	(.48)	(.52)	(.62)
N	11,825	11,825	4,670	11,825	11,825
Log likelihood	-351.46***	-350.39***	-277.56***	-355.03***	-357.33***

Note: Robust standard errors, with clustering on countries, in parentheses. Log-likelihood figures obtained from standard logistic regressions. * $p < 0.05$, ** $p < 0.01$, *** $p < 0.001$. $^{+}$ $p \leq 0.1$

Models A14-A17 in Table A7 replicate Models 4-7 of Table 3 in the main text, using the median, rather than the maximum, organizational scope value. Again, we find a direct effect of the scope variable on the risk of armed civil conflict onset whereas the interaction term of the variable with the logged number of other ethnic groups in the country remains insignificant. This provides further evidence that the effect of radical demands does not depend on the existence of potential future imitators, but results from an escalation in the dyadic government-movement interaction. Furthermore, the results of Table A7 suggest that governments' threat perception and the risk of violent conflict escalation are not driven solely by radical outliers within ethnic movements (i.e. the organizations with the maximum scope values), but that the scope of the demands made by movements' most "representative" organizations have an equally strong and systematic effect on armed civil conflict onset.

Table A7: Movement Demands and Ethnic Civil Conflict Onset. Robustness Tests II

	**Model A14**	**Model A15**	**Model A16**	**Model A17**
Scope of demands (median organizational value)	.97***<br>(.19)	.81***<br>(.19)	.98***<br>(.29)	.63*<br>(.27)
Scope of demands * N of other groups in country				.10<br>(.11)
N organizations (logged)	.35***<br>(.09)	-.01<br>(.10)	-.24*<br>(.12)	-.01<br>(.10)
Relative group size	.21<br>(.78)	.68<br>(.57)	.71<br>(.73)	.65<br>(.57)
N of TEK connections (logged)	.36*<br>(.15)	.47***<br>(.13)	.91***<br>(.22)	.48***<br>(.13)
Geographic concentration	.78<br>(.65)	.06<br>(.72)	.90<br>(.55)	.11<br>(.71)
Excluded		.54<br>(.34)	.98*<br>(.47)	.57<br>(.34)
Regional autonomy		.65*<br>(.31)	.92*<br>(.39)	.63*<br>(.30)
Downgraded in last 5 years		1.97***<br>(.52)	2.03***<br>(.51)	1.94***<br>(.53)
Liberal democracy		-.24<br>(1.06)	1.70<br>(1.33)	-.31<br>(1.06)
GDP per capita (logged)		-.12<br>(.16)	-.50<br>(.39)	-.10<br>(.17)
N of years of mobilization		.03**<br>(.01)	.06***<br>(.01)	.03**<br>(.01)
Use of small-scale violence by movement		1.91**<br>(.61)	2.10**<br>(.66)	1.88**<br>(.60)
Group’s conflict history		-.06<br>(.17)	-.13<br>(.24)	-.07<br>(.17)
N of other groups in country (logged)		.08<br>(.19)	1.38<br>(1.08)	.01<br>(.19)
Scope of demands of other groups		-.18<br>(.22)	-1.13*<br>(.57)	-.18<br>(.21)
Country population (logged)		-.17<br>(.11)	-.02<br>(.71)	-.15<br>(.10)
Calendar year		-.00<br>(.01)		-.00<br>(.01)
Cubic polynomial of peace years	Yes	Yes	Yes	Yes
Constant	-4.54***<br>(.55)	2.29<br>(21.01)	-6.82<br>(13.93)	3.22<br>(20.59)
Country-fixed effects	No	No	Yes	No
Year-fixed effects	No	No	Yes	No
N	11,825	11,723	4,930	11,723
Log likelihood	-351.40***	-295.42***	-210.93***	-295.26***

Note: Standard errors in parentheses. Clustering on countries in Models A14, A15, and A17, and on ethnic groups in Model A16.

*Log-likelihood figures obtained from standard logistic regressions. * p < 0.05, ** p < 0.01, *** p < 0.001.*

References in Appendix

Baetschmann, Gregori, Ballantyne, Alexander, Staub, Kevin E., and Winkelmann, Rainer. 2020. “feologit: A new command for fitting fixed-effects ordered logit models.” *The Stata Journal* 20(2): 253-275.
